# Supplementary material for: Regadenoson for the treatment of COVID-19: A five case clinical series and mouse studies
Source: PLoS One. 2023 Aug 11;18(8):e0288920. doi: 10.1371/journal.pone.0288920 (PMC10420352; doi:10.1371/journal.pone.0288920)
Supplement: S1 Table — (DOCX) [file pone.0288920.s002.docx]

**Supplemental Table 1. Data of Blood Pressure from 5 Patients**

**Systolic Blood Pressure (mmHg)**

| **Patient ID** | **101-001** | **101-002** | **101-003** | **101-004** | **101-005** |
| --- | --- | --- | --- | --- | --- |
| **Baseline** | **110** | **135** | **150** | **114** | **102** |
| **Post LD** | **112** | **131** | **133** | **116** | **99** |
| **4 hours into MD** | **115** | **104** | **133** | **102** | **102** |
| **6 hours into MD** | **112** | **ND** | **133** | **104** | **99** |
| **24 hours Post Infusion** | **136** | **113** | **124** | **117** | **106** |

**Diastolic Blood Pressure (mmHg)**

| **Patient ID** | **101-001** | **101-002** | **101-003** | **101-004** | **101-005** |
| --- | --- | --- | --- | --- | --- |
| **Baseline** | **74** | **75** | **85** | **76** | **56** |
| **Post LD** | **76** | **73** | **68** | **68** | **58** |
| **4 hours into MD** | **74** | **68** | **85** | **66** | **56** |
| **6 hours into MD** | **71** | **ND** | **78** | **63** | **59** |
| **24 hours Post Infusion** | **82** | **78** | **77** | **71** | **67** |

**LD: Loading dose, 5 µg/kg/h for 30 minutes. MD: Maintenance dose, 1.44 µg/kg/hour for 6 hours.**

**ND: Not detected.**
