# Supplementary material for: Regadenoson for the treatment of COVID-19: A five case clinical series and mouse studies
Source: PLoS One. 2023 Aug 11;18(8):e0288920. doi: 10.1371/journal.pone.0288920 (PMC10420352; doi:10.1371/journal.pone.0288920)
Supplement: S2 Table — (DOCX) [file pone.0288920.s003.docx]

Supplemental Table 2. Oxygen saturation (SpO2), D-Dimer and CRP data from the participant patients

SpO2

| Patient ID | Baseline | Post Infusion |
| --- | --- | --- |
| 101-001 | 95 | 100 |
| 101-002 | 95 | 98 |
| 101-003 | 92 | 96 |
| 101-004 | 93 | 95 |
| 101-005 | 94 | 94 |

D-Dimer (ng/ml)

| Patient ID | Baseline | Post Infusion |
| --- | --- | --- |
| 101-001 | 380 | 330 |
| 101-002 | 830 | 480 |
| 101-003 | 390 | 360 |
| 101-004 | 900 | 540 |
| 101-005 | 1270 | 880 |

CRP (mg/dL)

| Patient ID | Baseline | Post Infusion |
| --- | --- | --- |
| 101-001 | 0.5 | 0.5 |
| 101-002 | 3.3 | 1.4 |
| 101-003 | ND | ND |
| 101-004 | 4.1 | 2 |
| 101-005 | 7.3 | 4 |

ND: Not detected.
