# Supplementary material for: Regadenoson for the treatment of COVID-19: A five case clinical series and mouse studies
Source: PLoS One. 2023 Aug 11;18(8):e0288920. doi: 10.1371/journal.pone.0288920 (PMC10420352; doi:10.1371/journal.pone.0288920)
Supplement: S3 Table — (DOCX) [file pone.0288920.s004.docx]

Supplemental Table 3. Changes of CD69+ T cells, CD69+iNKT cells in the participated patients

CD69+ T cells (% of Total CD3+ T cells)

| Patient ID | Baseline | 30 Mins |
| --- | --- | --- |
| 101-001 | 5.11 | 2.74 |
| 101-002 | 12.09 | 19.07 |
| 101-003 | 2.74 | 4.54 |
| 101-004 | 8.6 | 6.4 |
| 101-005 | 15.97 | 12.8 |

CD69+ iNKT cells (% of total CD3+6B11+ iNKT cells)

| Patient ID | Baseline | 30 Mins | 4 Hours | 24 Hours |
| --- | --- | --- | --- | --- |
| 101-001 | 57.23 | 21.43 | 29.02 | 8.06 |
| 101-002 | 63.33 | 49.89 | 41.22 | 87.3 |
| 101-003 | 49.97 | 20.12 | 30.43 | 99.56 |
| 101-004 | 100 | 50.04 | 90.91 | 80.11 |
| 101-005 | 72.73 | 30.01 | 0 | 98.79 |
