# Supplementary material for: Regadenoson for the treatment of COVID-19: A five case clinical series and mouse studies
Source: PLoS One. 2023 Aug 11;18(8):e0288920. doi: 10.1371/journal.pone.0288920 (PMC10420352; doi:10.1371/journal.pone.0288920)
Supplement: S4 Table — (DOCX) [file pone.0288920.s005.docx]

**Supplemental Table 4. Levels of cytokine/chemokines (pg/ml) in the patient’s plasma**

| Patient ID | **MCP-1** | **IFN-G** | **IL-10** | **IL-12 P40** | **IL-12 P70** | **MIP-1A** | **IL-17** |
| --- | --- | --- | --- | --- | --- | --- | --- |
| 101-001-1 | 92.66 | 18.67 | 12.21 | 10.14 | 9.35 | 6.68 | 4.07 |
| 101-001-2 | 100.05 | 20.36 | 11.27 | 7.17 | 12.30 | 5.27 | 4.30 |
| 101-001-3 | 70.48 | 7.62 | 10.20 | 7.63 | 5.07 | 5.07 | ND |
| 101-001-4 | 113.41 | 11.75 | 10.09 | 9.16 | 7.85 | 5.87 | 0.69 |
| 101-002-1 | 31.34 | 2.79 | 22.24 | 6.16 | 1.98 | ND | ND |
| 101-002-2 | 28.36 | 1.81 | 19.18 | 4.61 | 1.36 | ND | ND |
| 101-002-3 | 35.57 | 2.14 | 17.86 | 6.84 | 1.59 | ND | ND |
| 101-002-4 | 34.61 | 1.81 | 14.50 | 5.13 | 2.04 | ND | ND |
| 101-003-1 | 40.59 | 2.47 | 15.73 | 7.67 | 2.72 | 1.63 | ND |
| 101-003-2 | 43.59 | 3.10 | 15.18 | 9.65 | 3.42 | 1.79 | 0.95 |
| 101-003-3 | 44.42 | 2.47 | 15.96 | 4.08 | 1.59 | 0.36 | ND |
| 101-003-4 | 59.70 | 3.10 | 15.86 | 5.13 | 1.16 | ND | ND |
| 101-004-1 | 47.46 | 9.19 | 19.62 | 20.96 | 7.96 | 27.36 | 5.91 |
| 101-004-2 | 53.62 | 9.97 | 18.96 | 24.16 | 10.54 | 24.18 | 8.26 |
| 101-004-3 | 58.90 | 5.30 | 12.55 | 13.03 | 5.71 | 24.09 | 3.02 |
| 101-004-4 | 60.66 | 5.86 | 18.30 | 12.55 | 5.38 | 21.75 | 1.45 |
| 101-005-1 | 53.77 | 4.56 | 24.38 | 23.16 | 4.21 | 14.69 | 4.96 |
| 101-005-2 | 70.06 | 3.35 | 21.00 | 20.85 | 2.22 | 11.23 | 4.71 |
| 101-005-3 | 75.15 | 5.28 | 27.48 | 28.75 | 3.88 | 17.02 | 9.60 |
| 101-005-4 | 85.03 | 8.40 | 52.55 | 19.09 | 4.38 | 13.48 | 3.20 |
|  |  |  |  |  |  |  |  |

| Patient ID | **IL-7** | **IL-1B** | **IL-2** | **TNF-A** | **IL-6** | **IL-8** | **IP10** |
| --- | --- | --- | --- | --- | --- | --- | --- |
| 101-001-1 | 4.33 | 2.30 | 0.78 | 7.84 | 6.62 | 15.79 | 91.60 |
| 101-001-2 | 3.15 | 1.56 | 0.72 | 7.57 | 7.07 | 15.04 | 88.55 |
| 101-001-3 | 0.95 | 1.25 | ND | 2.89 | 3.39 | 10.88 | 79.06 |
| 101-001-4 | 2.20 | 1.94 | ND | 4.65 | 4.92 | 12.45 | 77.98 |
| 101-002-1 | ND | 0.93 | ND | 0.48 | 1.45 | 14.69 | 13.60 |
| 101-002-2 | ND | 0.80 | ND | 0.56 | 1.99 | 10.13 | 12.53 |
| 101-002-3 | ND | 0.83 | ND | 0.30 | 2.25 | 15.86 | 12.40 |
| 101-002-4 | ND | 0.93 | ND | 0.30 | 2.33 | 7.94 | 15.05 |
| 101-003-1 | ND | 0.66 | ND | 0.81 | 1.81 | 6.13 | 32.56 |
| 101-003-2 | ND | 1.04 | ND | 1.12 | 3.39 | 5.86 | 38.92 |
| 101-003-3 | ND | ND | ND | ND | 4.25 | 6.46 | 25.10 |
| 101-003-4 | ND | 0.09 | ND | ND | 15.24 | 19.38 | 23.62 |
| 101-004-1 | 9.21 | 3.95 | 0.78 | 7.97 | 15.61 | 23.48 | 123.40 |
| 101-004-2 | 9.47 | 5.77 | 0.98 | 7.90 | 13.03 | 20.42 | 162.52 |
| 101-004-3 | 5.43 | 2.42 | ND | 5.95 | 13.21 | 18.13 | 67.83 |
| 101-004-4 | 11.81 | 2.18 | ND | 5.68 | 12.96 | 19.33 | 61.87 |
| 101-005-1 | 77.39 | 2.70 | 0.53 | 1.30 | 25.19 | 14.75 | 64.91 |
| 101-005-2 | 87.14 | 2.19 | ND | 1.05 | 26.75 | 14.37 | 55.28 |
| 101-005-3 | 171.47 | 3.91 | 0.53 | 1.84 | 44.31 | 21.38 | 113.55 |
| 101-005-4 | 39.03 | 1.21 | 0.43 | 1.00 | 24.94 | 11.93 | 43.68 |

ND: Not detected.
