# Supplementary material for: Regadenoson for the treatment of COVID-19: A five case clinical series and mouse studies
Source: PLoS One. 2023 Aug 11;18(8):e0288920. doi: 10.1371/journal.pone.0288920 (PMC10420352; doi:10.1371/journal.pone.0288920)
Supplement: S1 File — (PDF) [file pone.0288920.s006.pdf]

Introduction Page

1 * Abbreviated Title:
Regadenoson COVID-19 Trial

2 * Full Title:
Clinical Trial on the Safety and Efficacy of Regadenoson for Moderate to Severe COVID-19 Adult Patients.

3

* Select Type of Submission:

- (●) **IRB Application**
- ( ) Humanitarian Use Device (for FDA approved Indication & non-research purposes ONLY)
- ( ) Single Patient Expanded Access (pre-use)
- ( ) Single Patient Emergency Use (post-use)
- ( ) Unsure if this proposal requires IRB review (Not Human Subject Research)

Note: The Type of Submission cannot be changed after this application has been submitted for review.

4 Original Version #:
00

Research Team Information

1 * Principal Investigator - Who is the PI for this study (person must have faculty status)? ***Faculty status is defined as being a full-time (>51% effort) faculty member holding one of the following titles at UM: Professor; Associate Professor; Assistant Professor.***

Christine Lau

CITI Training:

1.1 * Does the Principal Investigator have a potential conflict of interest, financial or otherwise, related to this research?

○ Yes ● **No**

2 Point of Contact - Who is the alternative point of contact for the PI? This person can be a study coordinator or any other study team member. In case the IRB cannot contact the PI, this person is a secondary person to contact:

Emily Fleischmann

CITI Training: ID00010280

2.1 Does the Point of Contact have a potential conflict of interest, financial or otherwise, related to this research?

○ Yes ● **No**

3 Other Team Members - list all additional members of the research team for this study. DO NOT include the PI or POC in this list:

	Name	Edit Submission	cc on Email	Research Role	Has SFI?	CITI Training
View	Freshta Akbari	yes	yes	Research Team Member	no	ID00001608
View	Matthew Audette	yes	yes	Research Team Member	no	ID00010858
View	Melissa Culligan	yes	yes	Study Coordinator	no	ID00007152
View	Yunge Zhao	yes	yes	Research Team Member	no	
View	Manal Al-Suqi	yes	yes	Research Team Member	no	ID00006808
View	Joseph Rabin	no	no	Sub-Investigator	no	ID00008149
View	Ezzat Mostafa	yes	yes	Research Team Member	no	
View	Alexander Krupnick	no	no	Sub-Investigator	no	
View	Christopher Thomas	no	no	Research Team Member	no	ID00010729

IMPORTANT NOTE: All research team members (including PI) must have current CITI and HIPAA training completed.

Resources

If this study is a collaborative UM/VA study, please clarify which resources are being used at each institution.

1 * Describe the time that the Principal Investigator will devote to conducting and completing the research:

Dr. Lau is extremely devoted to the use of this drug in COVID-19 positive moderate to severe patients. Since she cannot enter the patient's room, she will actively be involved with the patient's COVID-19 physicians and will screen medical charts. She will spend 5-7 hours per week for this study.

2 * Describe the facilities where research procedures are conducted:

University of Maryland Medical Center

3 * Describe the availability of medical and/or psychological resources that subjects might need as a result of anticipated consequences of the human research:

1. Sufficient time to conduct, oversee and complete research - YES
2. Adequate number of qualified staff - YES
3. A process to ensure that all persons involved in the design, conduct and/or reporting of research are adequately informed about the protocol and their research-related duties and functions - YES
4. Adequate facilities in which to perform study procedures - YES
5. Availability of medical or psychological resources that participants may need as a consequence of the research - YES
6. Access to a population that will allow recruitment of the necessary number of participants - YES

4 * Describe the process to ensure that all persons assisting with the research are adequately informed about the protocol, the research procedures, and their duties and functions:

Dr. Lau is the creator of the protocol and grant. She will provide study personnel with adequate training in regards to the protocol, study procedures, and respective duties. All study staff are highly involved with the study and weekly meetings are held to update all members and discuss the study.

ID: VIEW4DF83CB976400
Name: v2_Resources

Sites Where Research Activities Will Be Conducted

1 * Is this study a:

- ○ Multi-Site
- ● **Single Site**

2 * Are you relying on an external IRB (not UM) to be the IRB of Record for this study?

○ Yes ● **No**

3 * Are any other institutions/organizations relying on UM to be the IRB of Record for this study?

○ Yes ● **No**

3.1 Attach the applicable regulatory documents here (i.e., IRB Authorization Agreement (IAA), FWA, local ethics approval, other IRB approvals, etc.). Final UM approval will be contingent upon final execution of all required regulatory approvals:

Name	Created	Modified Date

There are no items to display

4 * Is UM the Coordinating Center for this study? (Applicable for multi-site studies. A Coordinating Center is responsible for overall data management, monitoring and communication among all sites, and general oversight of conduct of the project.)

○ Yes ● **No**

5 Is VA the Coordinating Center for this study? (Applicable for Collaborative studies between the VA, UM and other sites. A Coordinating Center is responsible for overall data management, monitoring and communication among all sites, and general oversight of conduct of the project)

○ Yes ● **No**

6 * Institution(s) where the research activities will be performed:

- [x] **University of Maryland, Baltimore**
- [ ] University of Maryland, Upper Chesapeake Kaufman Cancer Center
- [ ] VAMHCS
- [x] **UMB School of Medicine**
- [ ] Marlene and Stewart Greenebaum Cancer Center
- [ ] University Physicians Inc.
- [x] **Shock Trauma Center**
- [ ] General Clinical Research Center (GCRC)
- [ ] Maryland Psychiatric Research Center (MPRC)
- [ ] Johns Hopkins
- [ ] International Sites
- [ ] UMB Dental Clinics
- [ ] Center for Vaccine Development
- [ ] Community Mental Health Centers
- [ ] Private Practice in the State of Maryland
- [ ] Institute of Human Virology (IHV) Clinical Research Unit
- [ ] Joslin Center
- [ ] UMB Student Classrooms
- [ ] National Institute of Drug Abuse (NIDA)

- [ ] National Study Center for Trauma and EMS
- [ ] Univ of MD Cardiology Physicians at Westminster
- [ ] Nursing Homes in Maryland
- [ ] University of Maryland Biotechnology Institute
- [ ] Maryland Department of Health
- [ ] Maryland Proton Treatment Center
- [ ] Mount Washington Pediatric Hospital
- [ ] Institute of Marine and Environmental Technology (IMET)
- [ ] Other Sites
- [x] **University of Maryland Medical System (Select below)**

* UMMS Sites:

- [x] **University of Maryland Medical Center**
- [ ] UMMC Midtown Campus (formerly Maryland General Hospital)
- [ ] UM St. Joseph Medical Center
- [ ] UM Baltimore Washington Medical Center
- [ ] UM Capitol Region Health
- [ ] UM Charles Regional Medical Center
- [ ] UM Shore Medical Center at Easton
- [ ] UM Shore Medical Center at Chestertown
- [ ] UM Shore Medical Center at Dorchester
- [ ] UM Shore Emergency Center at Queenstown
- [ ] UM Shore Regional Health
- [ ] University of Maryland Rehabilitation & Orthopaedic Institute (formerly Kernan Hospital)
- [ ] UM Upper Chesapeake Health
- [ ] UM Upper Chesapeake Medical Center
- [ ] UM Harford Memorial Hospital
- [ ] University of Maryland Community Medical Group

Funding Information

1 * Indicate who is funding the study:

- [x] **Federal**
- [ ] Industry
- [ ] Department / Division / Internal
- [ ] Foundation
- [ ] Private
- [ ] State Agency

2 * What portion of the research is being funded? (Choose all that apply)

- [x] **Drug**
- [ ] Device
- [x] **Staff**
- [ ] Participant Compensation
- [ ] Procedures
- [ ] Other

3 Please discuss any additional information regarding funding below:

This study was submitted to the NIH as a supplement to her RO1 grant from the NHLBI and picked up for funding, waiting for the IRB approval to finalize the funding process. FDA has granted an IND (IND 149635) for this study.

ID: VIEW4DF85DF452400
Name: v2_Funding Information

DHHS Funded Study

You indicated that this is a Federally funded study.

1 * Is this study sponsored by a Department of Health and Human Services (DHHS) agency?

● **Yes** ○ No

2 You may upload any grant documents here:

Name	Created	Modified Date
Funding notice letter from NIH(0.01)	8/4/2020 11:22 AM	8/4/2020 11:22 AM

ID: VIEW4DF87B9560800
Name: v2_DHHS Funded Study

Federal Agency Sponsor Contact Information

You indicated that this is a Federally funded study.

1 * Agency Name:
NIH-National Heart, Lung, and Blood Institute

* Address 1:
Building 31, 31 Center Drive

Address 2:

* City:
Bethesda, MD

* State:
MD

* Zip Code:
20892

* Contact Person:
Craig, Matt

* Phone Number:
1-877-645-2448

Grant Number 1 (if applicable):
- OR - Check here if Grant 1 is not assigned a number. ☑

If Grant 1 has no number, please provide the following information:
Title of Grant 1:
Treatment of Moderate to Severe COVID-19 Patients with Regadenoson
PI of Grant 1:
Christine Lau

Grant Number 2 (if applicable):
- OR - Check here if Grant 2 is not assigned a number. ☐

If Grant 2 has no number, please provide the following information:
Title of Grant 2:

PI of Grant 2:

Grant Number 3 (if applicable):
- OR - Check here if Grant 3 is not assigned a number ☐

If Grant 3 has no number, please provide the following information:
Title of Grant 3:

PI of Grant 3:

Grant Number 4 (if applicable):
- OR - Check here if Grant 4 is not assigned a number. ☐

If Grant 4 has no number, please provide the following information:
Title of Grant 4:

PI of Grant 4:

Research Protocol

1 * Do you have a research protocol to upload?

- (●) **Yes**
- ( ) No, I do not have a research protocol and will use the CICERO application to enter my study information

2 If Yes, upload the research protocol:

Name	Created	Modified Date
Lau-IND149635 Tracked changes 7.9.20 to 1.26.21.docx(0.01)	2/10/2021 10:29 AM	2/10/2021 10:29 AM
Lau-IND149635-3rd Submission_7.9.20.docx(0.01)	2/10/2021 10:21 AM	2/10/2021 10:21 AM
Lau-IND149635-Edits_1.26.2021 Clean.docx(0.01)	2/10/2021 8:46 AM	2/10/2021 8:46 AM

ID: VIEW4E00563F8D000
Name: v2_Research Protocol

Risk Level

What is the risk level of your study? (Ultimately, the IRB will determine the appropriate risk level and your designation is subject to change.)

* Choose One:

- ○ Minimal - The probability & magnitude of harm/discomfort anticipated in the research are not greater in and of themselves than those ordinarily encountered in daily life or during the performance of routine physical or psychological examinations/tests.
- ● **Greater Than Minimal - Does not meet the definition of Minimal Risk.**

ID: VIEW4E02805225800
Name: v2_Risk Level

Type of Research

1 * Indicate **ALL** of the types of research procedures involved in this study (Choose all that apply):

- [x] **Use of unapproved drug(s)/biologic(s) or approved drug(s)/biologic(s) whose use is specified in the protocol.**
- [ ] Evaluation of food(s) or dietary supplement(s) to diagnose, cure, treat, or mitigate a disease or condition.
- [ ] Use of device(s) whose use is specified in the protocol
- [ ] Psychological/Behavioral/Educational Method or Procedure (i.e., survey, questionnaires, interviews, focus groups, educational tests).
- [x] **Sample (Specimen) Collection and/or Analysis (including genetic analysis).**
- [x] **Data Collection or Record Review (i.e., chart review, datasets, secondary data analysis).**
- [ ] None of the above.

2 * Is this study a clinical trial OR will this study be registered at ClinicalTrials.gov?
A clinical trial is a research study in which one or more human subjects are prospectively assigned to one or more interventions (which may include placebo or other control) to evaluate the effects of those interventions on health-related biomedical or behavioral outcomes.

(●) **Yes** ( ) No

ID: VIEW4E0280569E000
Name: v2_Type of Research

Lay Summary

1 * Provide a summary of the background and purpose of the study in language that can be understood by a person without a medical degree.

More than 17 million people have been infected and 677K lives have been lost since the COVID-19 pandemic. Unfortunately, the effective treatments remain limited for this deadly virus. The moderate to severe COVID-19 patients suffer acute lung injury and need oxygen therapy, and even ventilators, to help them breathe. When a person gets a viral infection, certain body cells (inflammatory/immune cells) get activated and release a wide range of small molecules, also known as cytokines, to help combat the virus. But it is possible for the body to overreact to the virus and release an overabundance of cytokines, forming what is known as a "cytokine storm". When a cytokine storm is formed, these cytokines cause more damage to their own cells than to the invading COVID-19 that they're trying to fight. Recently, doctors and research scientists are becoming increasingly convinced that, in some cases, this is likely what is happening in the moderate to severe COVID-19 patients. The cytokine storm may be contributing to respiratory failure, which is the leading cause of mortality for severe COVID-19 patients. Therefore, being able to control the formation of cytokine storms will also help alleviate the symptoms and aid in the recovery of severe COVID-19 patients.

Our research team have discovered that stimulation of the A2A receptor, one of the molecules located on several inflammatory/immune cells, can decrease the amount of cytokines released, including IL-6, which is significantly increased in severe COVID-19 patients. Furthermore, our clinical trial showed that Regadenoson, an FDA approved drug that specifically targets A2A receptors, is able to inhibit the release of IL-6 as well as MMP-9, another molecule release by inflammatory/immune cells responsible for lung injury in lung transplantation patients. In addition, Regadenoson can also increase levels of TIMP-1, which is an endogenous inhibitor for MMP-9. We also found that Regadenoson had no obvious side effects in lung transplant patients.

We believe that Regadenoson treatment may be more effective than IL-6 inhibitor treatment in moderate to severe COVID-19 patients because it has three layers of functions (reducing IL-6, MMP-9, and increasing TIMP-1) to help prevent lung injury. As such, Regadenoson treatment may prove to be invaluable in reducing lung damage and saving the lives of severe COVID-19 patients before an effective vaccine is developed. Even in patients with milder symptoms, Regadenoson can help prevent further damage to their lungs, leading to fewer patients entering ICU and requiring ventilators.

ID: VIEW4E02805CF7000
Name: v2_Lay Summary

Justification, Objective, & Research Design

If you uploaded a separate research protocol document in the 'Research Protocol' page, cite the applicable section and page numbers from that document in the answer boxes below.

1 * Describe the purpose, specific aims, or objectives of this research. State the hypothesis to be tested:

We reason that Regadenoson treatment will reduce COVID-19-induced lung injury by inhibiting hyperinflammation. Our overarching goal is to demonstrate that Regadenoson treatment increases survival by reducing hyperinflammation and pulmonary function. We will test the hypothesis that Regadenoson elicits clinical improvement and enhances survival compared to placebo control patients with COVID-19.

Specific Aim 1: will determine the initial high dose followed by low dose continuous infusion that is safe and feasible in moderate to severe COVID-19 patients. Even if the dosages that we will use in moderate to severe COVID-19 patients have been proved to be safe in myocardial perfusion imaging patients, sickle cell disease, and lung transplantation patients, it is still unclear whether it is safe in COVID-19 patients. Therefore, our primary endpoint for this Aim will be safety. In cohort 1, we will be looking at any drug-related side effects and toxicity of Regadenoson as we did in lung Transplantation trial.

Specific Aim 2: will determine the potential efficacy of Regadenoson infusion in moderate to severe COVID-19 patients. If Regadenoson infusion is safe and feasible in the moderate to severe COVID-19 patients in Aim 1, we will test its efficacy in moderate to severe COVID-19 patients in a randomized controlled trial of Regadenoson versus placebo control. The primary endpoints of this specific aim are: 1) Proportion of patients alive and free of respiratory failure through the 30 day trial. Respiratory failure is defined based on resource utilization requiring at least 1 of the following modalities, 2) Endotracheal intubation and mechanical ventilation, 3) Oxygen delivered by high-flow nasal cannula (heated, humidified, oxygen delivered via reinforced nasal cannula at flow rates >20L/min with fraction of delivered oxygen ≥0.5), 4) Noninvasive positive pressure ventilation or CPAP, 5) ECMO.

Specific Aim 3: will explore the mechanisms of the effects of Regadenoson infusion in moderate to severe COVID-19 patients. If Regadenoson is proved to be effective in treating moderate to severe COVID-19 patients in Aim 2, we will continue our study in this Aim. We will measure 1) the plasma levels of Regadenoson in the collected blood samples (these will be done only in cohort 1 as we need specific time points; 2) the levels of pro-inflammatory cytokines (TNF-α, IL-1, IL-6, IL-12, IL-8, INF-γ, etc) and anti-inflammatory cytokines (IL-4 and IL-10), and 3) the levels of matrix metalloproteinase-9 (MM-9) and tissue inhibitor of metalloproteinase-1 (TIMP-1) in blood samples which will be collected from cohort 1 and 2 patients at baseline and post infusion day 1. In cohort 1, we will collect 2- additional study lab draws, one at the conclusion of the 30-min infusion and one at 4-hours into the 6-hours slow continuous infusion.

2 * Discuss the research design including but not limited to such issues as: probability of group assignment, potential for subject to be randomized to placebo group, use of control subjects, etc.:

Infusion of Regadenoson is safe when administered as a rapid (10 sec) bolus of 400 ug (about 6.66 ug/kg body weight) for myocardial perfusion imaging (MPI) or when infused at a rate of 1.44 ug/kg/h for 12 h to patients with sickle cell anemia or following lung transplantation. Our primary goal is to determine if Regadenoson is safe and well tolerated when administered to hospitalized COVID-19 patients. Secondary endpoints will examine evidence of efficacy in order to make a go: no go determination for a subsequent clinical trial.

In cohort 1, we will enroll patients to test the safety, tolerability and toxicity in moderate to severe COVID-19 patients. Initial subjects in cohort 1 will receive a loading dose of 5.0 µg/kg (not to exceed 400 µg) over 30 minutes followed by a low dose infusion of 1.44 µg/kg/hr. This dose is somewhat less than, and is administered more slowly than the 6.66 ug/kg routinely used for MPI. If two dose limiting toxicities occur at a loading dose of 5.0 µg/kg then the loading dose will be halved to 2.5 µg/kg and enrollment will continue. If two dose limiting toxicities occur at a loading dose of 2.5 µg/kg then the Data and Safety Monitoring Board (DSMB) will convene to determine if the study should continue and omitting the loading dose may be considered. Alternatively, the DSMB will convene after 5 out of 5 patients or 5 out of 6 patients tolerate a given loading dose to make a determination on moving forward to cohort 2.

Cohort 2 will be randomized, double-blind, and placebo controlled. The randomization ratio is 1:1, Regadenoson to placebo (saline). Standard of care will be followed for all patients enrolled.

Described on page 7 IND version 1.26.2021

3 * Describe the relevant prior experience and gaps in current knowledge. Describe any relevant preliminary data:

The adenosine 2A receptor (A2AR) is expressed on immune cells (lymphocytes, neutrophils, macrophages, dendritic cells, T cells, B cells, NK cells and platelets) and its activation by adenosine or synthetic A2AR agonists results in a range of anti-inflammatory responses. Synthetic A2AR agonists have tissue-protective effects in inflammatory diseases. At the cellular level A2AR agonists have been shown to decrease the production of many anti-inflammatory cytokines; decrease neutrophil, platelet and endothelial cell activation, and decrease neutrophil adherence to endothelial cells. We found that the rank order potency of A2A agonists to bind to A2A receptors as assessed by radioligand binding parallels their potency to inhibit neutrophil oxidative burst and to elevate cyclic AMP production in live cells. These studies and many others have established that agonists of A2AR hold promise for mitigating acute inflammatory conditions. In addition to inhibiting the production of pro-inflammatory cytokines, A2AR activation has been implicated in the induction of anti-inflammatory cytokines. For example, increased A2AR expression leads not only to inhibition of pro-inflammatory IL-12 secretion, but markedly upregulates anti-inflammatory IL-10 secretion by macrophages. Similarly, we found that A2AR agonists such as Regadenoson and Apadenoson modulate NKT-dendritic cell interactions to suppress IFN-gama and enhance IL-10 expression in liver and kidney IRI. Taken together these data suggest that regulation of innate immune cells by A2AR activation is critical for the termination of inflammatory responses.
Lethality of COVID-19: Lethality due to the virus is associated with pulmonary hyper-inflammation. Accumulating evidence suggest that a critically ill subgroup of patients with COVID-19 have cytokine storm syndrome. Predictors of lethality from a multicenter study of 150 confirmed COVID-19 cases in Wuhan, China, included elevated IL-6, consistent with the idea that mortality might be due to virally driven hyper-inflammation. Reducing cytokines is expected to have a beneficial effect during cytokine storm. Data from a phase 3 randomized controlled trial of IL-1 blockade (Anakinra) in sepsis, showed significant survival benefit in patients with hyper-inflammation without increased adverse events. Our preliminary data show that Regadenoson infusion reduces IL-6 production in recipients of transplanted lungs.
We expect that A2A agonists will be useful for the treatment of patients with virally induced (specifically SARS-CoV-2-induced) lung inflammation and dysfunction. A2A agonists also produce platelet and vascular effects that may also be beneficial by reducing blood pressure and coagulation.
Preliminary Data: A2AR activation attenuates lung inflammation and injury during acute lung injury (ALI) and ischemia reperfusion-injury (IRI). We demonstrated that activation of A2ARs on myeloid cells attenuates cytokine release and recruitment/adhesion of neutrophils to pulmonary endothelial cells following LPS-induced lung injury. IRI triggers a rapid and robust inflammatory response that provokes rapid release of pro-inflammatory cytokines, chemokines, and danger signals. A critical step is engagement of neutrophils with injured vascular endothelium, resulting in release of destructive proteases, oxygen radicals and cytokines that promote injury; ultimately leading to endothelial/epithelial barrier disruption, edema, impaired gas exchange, and lung failure. These studies have demonstrated the ability of A2AR agonists to attenuate the rapid production of proinflammatory cytokines, such as TNF-gama, IL-17, and IL-6. Extensive collaborative studies among our laboratories over the past 15 years have firmly established that A2AR agonists significantly protect lungs from IRI in rabbits, rats, and mice. Moreover, treatment with an A2AR agonist improved lung function and attenuated lung injury, edema, and cytokine production in a pig lung transplant model. Taken together, these animal studies provide strong evidence that A2AR agonists are attractive targets for therapeutic intervention in lung injury.

A2A agonists reduce mortality in models of bacterial sepsis: Our recent unpublished data demonstrate that ATL-146e and several other A2A agonists greatly reduced lethality in mouse and rat models of sepsis. Furthermore, these compounds are most effective when combined with antibiotics. These findings suggest that the protective effects of A2AR activation may be most prominent when the immune system becomes overactive.
Preliminary data from Regadenoson clinical trials: Our ongoing phase I trial in lung transplant patients showed that infusion of Regadenoson inhibits IL-6 and MMP-9 production when compared to controls. A2AR agonists also were found to inhibit the activation of iNKT cells during sickle cell pain crises. iNKT cells are key for initiating an inflammatory cascade.

Described on page 14-18 IND version 1.26.2021

4 * Provide the scientific or scholarly background, rationale, and significance of the research and how it will add to existing knowledge:

Recent studies demonstrate that respiratory failure from ARDS is the leading cause of death by COVID-19. Critically ill patients exhibit cytokine storm syndrome defined as elevated levels of various pro-inflammatory cytokines, including IL-6, TNFα, and IFN-γ. Cytokine storm contributes to lung inflammation and impaired pulmonary function. Activation of adenosine 2A receptors (A2AR) inhibits pro-inflammatory cytokine release and decreases endothelial adhesion molecule expression during acute tissue injury or transplantation in various animal models (mouse/rat/pig). Our published and unpublished data show that A2AR agonists reduce lethality and cytokine storm associated with bacterial sepsis in mouse and rat models. Our phase I trial in lung transplant patients shows that infusion of Regadenoson inhibits IL-6 production. Regadenoson also decreases matrix metalloproteinase-9 (MMP-9) which plays an important role in acute lung injury by increasing vascular permeability and promoting inflammatory cell extravasation. We propose that Regadenoson be used for the treatment of hamsters and patients with virally-induced lung hyper-inflammation. We hypothesize that A2AR agonist treatment will decrease pulmonary cytokine storm evoked by virus, reduce lung inflammation, and enhance pulmonary function. The combination of Regadenoson and Remdisivir may prove to be synergistic and we will check this in the hamster model. A2A agonists also have been reported to have potentially beneficial renal-protective and anti-coagulant effects.

This study is based on the innovative use of the FDA approved A2AR agonist, Regadenoson. Another notable aspect of this proposal is that it exploits extensive preclinical work and our ongoing phase I trial findings in lung transplantation. The principal goals of this proposal are: 1) to prove that regadenoson is safe in COVID-19 patients; and 2) to accrue enough efficacy data to make a “go: no go” decision for a definitive clinical trial.

There are several innovative aspects of this proposal: 1) it exploits our decades of pre-clinical research into how adenosine limits acute tissue injury. 2) It builds on our phase I trial of Regadenoson for lung transplantation. 3) Regadenoson is unusual in that it influences the production of many cytokines, and is not limited, like many other agents currently under investigation, to targeting a single cytokine or cytokine receptor. 4) In addition to inhibiting cytokine production, Regadenoson may provide additional benefits for severe COVID-19 patients by inhibiting coagulation, vascular leak and MMP-9 release. 6) In addition, for SARS-CoV-2, Regadenoson may prove to be effective for the treatment of SARS caused by other pathogens.

Supporting Literature

1 * Provide a summary of current literature related to the research: ***If you uploaded a separate research protocol document in the 'Research Protocol' page, cite the applicable section and page numbers from that document in the answer box below.***

The newly published data showed that respiratory failure from acute respiratory distress syndrome (ARDS) is the leading cause of mortality for severe COVID-19 patients. These patients had a cytokine storm syndrome, as indicated by elevated levels of various proinflammatory cytokines, including IL-6, TNFα, IFN-γ and others. Therefore, there is an urgent need to identify and treat hyperinflammation using existing, approved therapies with proven safety profiles to address the immediate need to reduce the mortality of moderate to severe COVID-19 patients. Our laboratories and others have showed that activation of adenosine A2A receptor (A2AR), using A2AR agonists, reduced lung acute injury by inhibiting pro-inflammatory cytokines in several animal models. Our ongoing phase I trial demonstrated that Ragadenoson (an A2AR agonist) treatment not only inhibited pro-inflammatory cytokines and matrix metalloproteinase-9 (MMP-9), but also increased the levels of TIMP-1 (an endogenous inhibitor of MMP-9) and anti-inflammatory cytokine (IL-10) in lung transplantation patients. MMP-9, secreted by several inflammatory cells (including neutrophils and macrophages), played an important role in acute lung injury by increasing vascular permeability and promoting inflammatory cell infiltrating into the lungs. Ragadenoson is a FDA approved drug and has been proved to be safe in our ongoing trial and in adult sickle cells patients. Based on these evidences, we propose a short clinical study of 40 moderate to severe Covid-19 patients to seek evidence that Regadenoson is effective in reducing lung inflammation and/or reducing mortality. This proposal is trying to expand our ongoing phase I trial in lung transplantation to treat moderate to severe Covid-19 patients.

Please also see References on page 10, 18-20 IND version 1.26.2021

2 If available, upload your applicable literature search:

Name	Created	Modified Date
Literature list (0.01)	8/4/2020 11:35 AM	8/4/2020 11:35 AM

Study Procedures

If you uploaded a separate research protocol document in the 'Research Protocol' page, cite the applicable section and page numbers from that document in the answer boxes below. (If this study is a collaborative UM/VA study please list each procedure that is being conducted and the locations where it is being conducted.)

1 * Describe all procedures being performed for research purposes only (these procedures would not be done if individuals were not in the study) and when they are performed, including procedures being performed to monitor subjects for safety or to minimize risks:

Study product infusion:
Cohort 1 will receive Regadenoson. Cohort 2 will be randomized 1:1 (regadenoson:placebo). When given, Regadenoson will be given intravenously as a 5 ug/kg loading dose over 30 mins (to avoid unpleasant side effects sometimes associated with the rapid bolus injection of Regadenoson), followed by a continuous slow infusion (1.44micrograms/kg/hour) with the use of a pediatric infusion pump for 6 hours. Please see research design section that describes potential lower doses due to dose limiting toxicities.An equivalent volume of saline will be given to patients receiving the placebo.

Blood draws:
In cohort 1 blood will be drawn for research purposes at the following 4 timepoints: prior to infusion, at the conclusion of the 30min load dose, 4 hours into the slow infusion, and day 1 post infusion. In cohort 2, 2 blood draws will be obtained for research once prior to infusion and once on post infusion day 1. Blood samples will be used to evaluate the plasma levels of regadenoson, cytokines, cells, and enzymes. 10 ml blood will be collected at each time point.

PT, PTT, INR, platelets, and TEG labs will be drawn prior to infusion and on post infusion day 1.

Cardiac telemetric monitoring during the infusion of Regadenoson, and up to 4-hours after the conclusion of the drug infusion and cardiac markers only if cardiac symptoms occur. Cardiac monitoring may be standard of care depending on clinical status of the patient.

Pregnancy test for patients of child bearing potential as needed for screening.

See p. 7, 21, 22, 41 of IND version 1.26.2021

2 * Describe all procedures already being performed for diagnostic or treatment purposes (if not applicable to the study, enter "N/A"):

Screening:
COVID-19 test, medical history/demographics, medications, physical exam with vitals, labs if available(CBC w/diff, CMP, sedimentation rate, creatine kinase, ABG), chest imaging, EKG

Day 1, 3, 7, 15, 30:
vitals, physical exam, labs (CBC w/diff, CMP, sedimentation rate, creatine kinase, ABG), chest imaging, EKG. All assessments recorded if available from standard of care records.

See p. 21, 22, 41 of IND version 1.26.2021

3 * Describe the duration of an individual participant's participation in the study:

30 days post Regadenoson infusion for each subject.

4 * Describe the amount of time it will take to complete the entire study:

Approximately 14 months.

5 * Describe any additional participant requirements:

N/A

Sample Size and Data Analysis

If you uploaded a separate research protocol document in the 'Research Protocol' page, cite the applicable section and page numbers from that document in the answer boxes below.

1 * Provide the rationale and sample size calculations for the proposed target population:

This trial will provide estimates of outcome variability in the primary and secondary outcomes that will be valuable for planning a larger randomized trial. Additionally, in planning for a larger trial, exploratory analyses will adjust estimates of treatment differences by known risk factors. Although we recognize that these analyses will be limited by the number of observed events, they will be useful for decisions on eligibility criteria or stratification factors in the larger trial. The analyses will be carried out on data from all randomized participants.

We will enroll 40 moderate to severe COVID-19 patients, which is defined in the inclusion Criteria above. Due to the mixture of the moderate to severe COVID-19 patients with various baseline severity levels, we will record any information regarding each patient, such as age, sex, symptoms, vital signs, saturation of oxygen, medications, treatments, days of illness, comorbidities, chest X-ray imaging, and baseline severity (such as, whether on oxygen, on ventilator, or on ECMO etc.) prior to Regadenoson infusion. When we analyze the effects of Regadenoson on COVID-19, we can subgroup these patients and conduct primary efficacy analysis and propose appropriate methods of covariate adjustment. For each subject, we will clearly document the reason for hospital admission, document the standard of care followed for each subject, and if care decisions are made based on resource limitation.

Up to 45 subjects may consent to the study to allow for withdrawals or failure to treat, however 40 subjects will be treated.

Please also see p. 8-10 IND version 1.26.2021

2 * Provide the plan for data analysis. Include in the description the types of comparisons that are planned (e.g., comparison of means, comparison of proportions, regressions, analysis of variance, etc.), which is the primary comparison/analysis, and how the analyses proposed will relate to the primary purposes of the study:

Please see p 8-10 IND version 1.26.2021

The primary outcome is the proportion of patients alive and free of respiratory failure through the 30-day trial. The total sample size of 40 participants will be randomized equally between the usual care and Regadenoson groups, using a randomly permuted block design, with random block sizes of 2 and 4. This plan will be generated by the study statistician. Within each group, the primary outcome proportion can be estimated with a standard error of no more than 11 percentage points; the difference between the groups can be estimated with a standard error that is no more than 16 percentage points. These calculations are based on the most conservative estimate in terms of standard error, using a proportion of 50% in each group. If, as expected, the proportions in each group are greater, such as 80% in usual care and 90% in the Regadenoson group, the difference can be estimated with a standard error of 11 percentage points. While these are relatively large standard errors, they are adequate to meet the objective suggested by the IND review, providing estimates of outcome proportions to be used in a larger trial.

To assess safety, the DSMB statistician will tabulate sequentially the proportion of participants in each group who unexpectedly experience serious cardiovascular side effects or persistent (> 30 min) intolerable side effects, based on clinical experience with Regadenoson. Intolerable side effects include headache or dyspnea. If the lower bound of a one-sided 80% confidence interval for the difference in proportions exceeds 10%, we will pause the trial, examine the adverse event profiles, and consider modifying dosing. With this rule, if the true proportion of patients who experience serious side effects is 20% in each group, there is a 16.5% chance that the trial will be paused; if the true proportion with Regadenoson is 40%, there is a 58% chance the study will be paused.

To plan for a future study, we will compute a 90% one-sided confidence interval (CI) for the difference in failure free survival (FFS) proportions at 30 days. The CI will use standard formulas for estimates and standard errors for the Kaplan-Meier estimate of the FFS distribution at a specific point in time (Klein et al, 2007). Censoring on the primary outcome will be unlikely since we will have follow-up at 30 days for FFS. For the secondary outcome of time to improvement as well as for the length of stay outcomes, survival time will be used as a competing risk (see Brock et al, 2011 and Harhay et al, 2019). Both of these papers recommend the use of cumulative incidence curves (CIC) for competing risks, as described by Kleinbaum and Klein, 2012). Group comparisons of the specific CIC can be made with the test of Grey (1988); with covariates, the method of Fine and Gray (1999) can be used to model the CIC.

Sharing of Results

1 * Describe whether results (study results or individual subject results, such as results of investigational diagnostic tests, genetic tests, or incidental findings) will be shared with subjects or others (e.g., the subject's primary care physicians) and if so, describe how it will be shared:

Study results or individual subject results will be shared as necessary to protect subject safety.

ID: VIEW4E02808CBD800
Name: v2_Sharing of Results

Research with Drugs or Biologics

You indicated on the "Type of Research" page that your study involves use of unapproved drug(s)/biologic(s) or approved drug(s)/biologic(s) whose use is specified in the protocol AND/OR evaluation of food(s) or dietary supplement(s) to diagnose, cure, treat, or mitigate a disease or condition.

1 * List all drugs/biologics to be administered in this study. Be sure to list each drug/biologic with its generic name only.

	Drug Name	FDA Approved	IND Number	PI IND Holder
View	Regadenoson	yes	149635	yes

2 * Attach the drug package insert or investigational drug brochure for the drugs being administered in this study:

Regadenoson(0.01)	4/28/2020 10:33 AM	4/28/2020 10:33 AM

3 If more than one drug is administered, discuss the risk implications of drug/therapy interactions:

4 * Will you be using Investigational Drug Services?

● **Yes** ○ No

Placebos

1

* Is this study placebo controlled?

● **Yes** ○ No

ID: VIEW4E0514EECCC00
Name: v2_Placebos

Placebo Use

You indicated that this study is placebo-controlled.

If you uploaded a separate research protocol document in the 'Research Protocol' page, cite the applicable section and page numbers from that document in the answer box below.

1.1 * Justify the use of the placebo study design and how the benefit to society outweighs the risks to the participants:

Activation of adenosine 2A receptors inhibits inflammation by directly targeting multiple inflammatory/immune cells. It is well established that the activity of inflammatory cells, including invariant natural killer T (iNKT), NKs, macrophages, DCs, monocytes, T-cells, platelets, and neutrophils, is inhibited by A2AR activation, resulting in reduced proinflammatory cytokines and decreased endothelial adhesion molecule expression during acute lung injury in different animal models (mouse/rat acute lung injury models, pre-clinical porcine lung transplantation model) [1-4]. A2AR agonist inhibited inflammatory/immune cells (such as iNKT cells and neutrophil) activation and infiltration into the lungs, Therefore, A2A agonists reduce acute lung injury through inhibiting proinflammatory cytokine releasing (including IL-6, TNFα, IFN-γ. etc) [5-9]. Our unpublished data showed A2A agonists also have been found to reduce lethality to cytokine storm associated with bacterial sepsis (Figures 1 & 2). Our phase I trial in lung transplant patients showed that low dosage, slow infusion of Regadenoson inhibited IL-6 production when compared with a patient without Regadenoson treatment (Figure 3). Regadenoson also decreased matrix metalloproteinase -9 (MMP-9) levels, but not MMP-2 level, when compared with control patient (Figure 4). We demonstrated that MMP-9, but not MMP-2, was significantly elevated in the acute injured lungs when compared with sham operated lungs [10]. The recent publications showed that MMP-9 plays an important role in acute lung injury by increasing vascular permeability, promoting inflammatory cell infiltrating into the lungs and positively associating with IL-6, TNFα, and IL-8 [11-14]. In addition, our proteomic analysis data showed that Regadenoson treated lung transplantation patients increased tissue inhibitor of metalloproteinase -1 (TIMP-1) when compared with control patient at the same time points (Figure 5). TIMP-1 is an endogenous inhibitor of MMP-9. Therefore, Regadenoson not only down-regulated MMP-9, but also up-regulated it's inhibitor. The phase II trials of adult sickle cell patients showed that Regadenoson inhibited iNKT cells, which is a key immune cell involved in both innate and adaptive immune response [15]. The newly published data showed that respiratory failure from acute respiratory distress syndrome (ARDS) is the leading cause of mortality for severe COVID-19 patients. The severe COVID-19 patients might have a cytokine storm syndrome, as indicated by elevated levels of various proinflammatory cytokines, including IL-6, TNFα, IFN-γ and others [16-29]. These life-threatening cytokines impair pulmonary function. Therefore, there is an urgent need to identify and treat hyperinflammation using existing, approved therapies with proven safety profiles to address the immediate need to reduce the high 2-4% mortality of severe COVID-19 patients. We propose that Regadenoson be used for the treatment of patients with virally induced lung hyperinflammation. We predict that A2AR agonist treatment will decrease the pulmonary cytokine storm evoked by viruses, reduce lung inflammation and enhance pulmonary function. Based on our current phase 1 trial data and published evidence, we expect that Redadenoson may save lives of severe COVID-19 patients through several different mechanisms (inhibiting proinflammatory cytokine (IL-6) and MMP-9 and increasing TIMP-1).

Please see page 14-15 IND version 1.26.21

1.2 * Is the placebo being used in place of standard therapy?

○ Yes ● **No**

1.3 * Is the standard treatment considered effective?

● **Yes** ○ No

Sample Collection/Analysis

You indicated on the "Type of Research" page that your study involves a sample (specimen) collection and/or analysis.

1 * What type of samples will be involved in this study? (Check all that apply)

- [x] **Prospective (will be collected)**
- [ ] Existing (previously collected at the time of initial IRB submission)

2 * Will genetic analysis/testing be done on any of the samples?

○ Yes ● **No**

3 * Will this study involve banking of samples (storing for future research use)?

○ Yes ● **No**

4 * What is the purpose of the sample collection and/or analysis?

To measure the plasma level of Regadenoson, cytokines, cells, and enzymes in the blood.

5 * Is there the possibility that cell lines will be developed with any of the samples?

○ Yes ● **No**

6 * Will the samples be released to anyone not listed as an investigator on the protocol?

● **Yes** ○ No

6.1 If Yes, give name(s) and affiliation(s):

The blood samples drawn for research purposes only may be analyzed at the University of Virginia and/or the Translational Core Lab.

7 * Will the sample material be sold or given to any third parties?

● **Yes** ○ No

7.1 If Yes, give name(s) and address(es):

The blood samples drawn for research purposes only may be analyzed at the University of Virginia and/or the Translational Core Lab.

Prospective Samples

You indicated that the study involves collection of prospective samples (specimens).

1 * What type of sample will be collected? (Check all that apply)

- [x] **Blood**
- [ ] Bone Marrow Aspirate/Biopsy
- [ ] Cerebrospinal Fluid
- [ ] Saliva
- [ ] Skin
- [ ] Sputum
- [ ] Stool
- [ ] Tissue
- [ ] Tumor
- [ ] Urine
- [ ] Other

1.1 If Other, specify:

2 For blood draws, specify the amount drawn, in teaspoons, at each visit and across the course of the subject's entire participation time:

Blood samples will be collected from cohort 1 COVID-19 patients prior to the infusion, after the 30 min load dose, 4 hours into the slow infusion, and on post infusion day 1. Each sample will be 10 ml for a total of 40ml or 8 teaspoons

Blood samples will be collected from the cohort 2 COVID-19 patients prior to the infusion and on post infusion day 1. Each samples will be 10ml of blood for a total of 20 ml or 4 teaspoons

3 * What type of samples will be collected? (Check all that apply)

- [x] **Samples obtained specifically for research purposes-obtained via a separate collection procedure done solely for the purposes of the study**
- [ ] Samples obtained specifically for research purposes-additional taken during a clinical procedure
- [x] **Leftover samples that were obtained for clinical purposes (no additional research procedures required)**
- [ ] Commercial (for profit) samples
- [ ] Other

3.1 If Other, specify:

4 * How are these samples labeled? For example, do they contain name, initials, dates, Social Security number, medical record number, or other unique code?

Standard of care labs will be labeled and processed according to the institutional procedures.

The blood drawn for research purposes will be labeled with the subject ID and will not include patient identifiers.

5 * Will sample(s) be made available to the research subject (or his/her medical doctor) for other testing?

○ Yes ● **No**

6 * If a participant withdraws from the study, will that participant have the option to get the remaining portion of their sample(s) back?

○ Yes ● **No**

7 * If the participant withdraws, explain how their sample(s) will be handled (For example, will sample(s) be destroyed, anonymized, etc.):

Samples from withdrawn patients will be destroyed if not already analyzed.

8 * Will the samples be destroyed after the study is over?

● **Yes** ○ No

8.1 If No, describe how the samples will be stored, where they will be stored, and for how long.

ID: VIEW4E0E257D60C00
Name: v2_Prospective Samples

Data Collection/Record Review

You indicated on the "Type of Research" page that your study involves data collection or record review (i.e., chart review, not self-report).

1 * What type of data will be collected/analyzed in this study? (Check all that apply)

- [ ] Retrospective/Secondary Analysis (data has already been collected at the time of initial IRB submission)
- [x] **Prospective (data is not yet in existence and/or collected)**

2 * Will this study involve adding data to a registry or database for future use?

○ Yes ● **No**

3 * Will the data be released to anyone not listed as an investigator on the protocol?

○ Yes ● **No**

3.1 If Yes, give name(s) & affiliation(s):

Prospective Data

You indicated that the study involves the collection of prospective data.

1 * Where is the data being collected from? (Check all that apply)

- [x] **Medical records**
- [x] **Medical images**
- [ ] Commercial (for profit) entity
- [ ] Publicly available records
- [ ] Schools
- [ ] Other

1.1 If Other, please specify:

2 * What data fields will you have access to/collect for the study? For example, name, initials, date of birth, Social Security number, income, demographic information, family units, housing, etc.

Medical records will be reviewed to collected clinical data and demographics. This includes age, date of birth, MRN, sex, race, symptoms, signs, saturation of oxygen, medications, treatments, days of illness, comorbidities, chest X-ray imaging, additional COVID treatments and baseline severity.

Please see pg. 41 of IND version 1.26.21

You can also upload a copy of the data fields/variables to be collected for the study:

Name	Created	Modified Date
There are no items to display		

Clinical Trial Registration

You indicated on the "Type of Research" page that your study is a clinical trial.

1 * Does the UM Clinical Trials Registry policy require registration of this trial?

- (selected) **Yes**
- No

2 * Has this trial been registered?

- (selected) **Yes**
- No

ID: VIEW4E093BF078C00
Name: v2_Clinical Trial Registration

Clinical Trial Registration Information

You indicated that this clinical trial has been registered.

1 * Was this trial registered at www.clinicaltrials.gov?
(●) **Yes** ( ) No

2 If no, was this trial registered on a site other than clinicaltrials.gov?
( ) Yes ( ) No

2.1 If Yes, specify the name of the other site:

2.2 Provide justification for registering this trial on this site:

3 * Registration Number
NCT04606069

Participant Selection

1 * How many local potential participants (or specimens/charts) do you anticipate will be screened for this study? ***Screening includes determining potential participants' initial eligibility for and/or interest in a study.***

110

2 * How many participants (or specimens, or charts) will be enrolled/used for this study? ***A local prospective participant is considered enrolled in the study when a UM-approved Informed Consent Document (not including separate screening consent forms) is signed.***

Local - the number being enrolled at this site:

45

Worldwide - the number being enrolled total at all sites (including local enrollment):

45

3 * Gender:

- [x] **Male**
- [x] **Female**

4 * Age(s):

- [ ] 0 to 27 days (newborn infants)
- [ ] 28 days to 12 months (Infant)
- [ ] 13 months to 23 months (Toddler)
- [ ] 2 to 5 years (Preschool)
- [ ] 6 to 11 years (Child)
- [ ] 12 to 17 (Adolescents)
- [x] **18 to 88 years (Adult)**
- [x] **89 years and older**

5 * Race/Ethnicity:

- [x] **All Races Included**
- [ ] American Indian or Alaskan Native
- [ ] Asian/Other Asian
- [ ] Asian/Vietnamese
- [ ] Black or African American
- [ ] Hispanic or Latino
- [ ] Mixed Race or Ethnicity
- [ ] Native Hawaiian or Pacific Islander
- [ ] White or Caucasian

6

* Language(s):

- [x] **English**
- [ ] Chinese
- [ ] French
- [ ] Italian
- [ ] Japanese
- [ ] Korean
- [ ] Local Dialect

- [x] **Spanish**
- [ ] Vietnamese
- [ ] Other

6.1 Specify Other:

7 * Are you excluding a specific population, sub-group, or class?

○ Yes ● **No**

7.1 If Yes, indicate your justification for excluding a specific population, sub-group, class, etc.:

ID: VIEW4E0E519C1D000
Name: v2_Participant Selection

Vulnerable Populations

1 * Will you be targeting ANY of the following Vulnerable Populations for enrollment? (Select all that apply)

- [x] **Employees or Lab Personnel**
- [ ] Children (Minors)
- [x] **Cognitively Impaired/ Impaired Decision Making Capacity**
- [ ] Pregnant Women/Fetuses
- [ ] Wards of the State
- [x] **Students**
- [ ] Prisoners
- [ ] Nonviable Neonates or Neonates of Uncertain Viability
- [x] **Economically/Educationally Disadvantaged**
- [ ] None of the above

Only select populations which you will be targeting for enrollment. Do not include populations that may be enrolled incidentally. Enrollment of a vulnerable population is considered to be "targeted" if the study team will be aware that a subject is from a vulnerable group as a result of interaction with the subject or collection of specific information about the subject, and the research team does not wish to exclude them. "Incidental" enrollment is limited to situations where a study team is unaware that a subject is from a vulnerable group.

Vulnerable Populations - Employees or Lab Personnel

You indicated that employees or lab personnel are included in this study.

1 * Describe how you will ensure particiption in this research will not affect employment and prevent undue influence:

In the event that employees or lab personnel are eligible for the study they will be approached about the study. It will be stated that their choice regarding study participation will not impact their employment or position at the institution. Consent will proceed as described in the consent section.

ID: VIEW4E0E5192BA800
Name: v2_Vulnerable Populations - Employees or Lab Personnel

Vulnerable Populations - Cognitively Impaired

You indicated that individuals who are cognitively impaired are included in this study.

1 * Describe how you will prevent undue coercion:

Due to the severity of their condition, patients eligible for this study may not be able to provide consent. Informed consent may be obtained from a legally authorized representative (LAR). It will be clearly stated that the research is voluntary and denial will have no impact on the care of the patient. The LAR will be given ample time to consider the study and ask questions. All questions will be answered adequately. A copy of the signed consent form will be given to the LAR.

2 * How will the capacity of these individuals to provide informed consent be assessed? How will you determine the need for a legally authorized representative?

We will utilize an Evaluation to Sign Consent Form. If the patient is unable to answer questions within this form, we will make the determination that the patient is unable to consent for themselves. We will then approach their LAR for consent.

You can also upload a copy of the tool that will be used to evaluate capacity:

Name	Created	Modified Date
Eval to Sign Consent Form.pdf(0.01)	9/16/2020 5:38 PM	9/16/2020 5:38 PM

3 * From which participants, who are not able to provide legally effective informed consent, will assent be obtained?

- ○ All participants
- ○ Some participants
- ● **None of the participants**

Vulnerable Populations - Students

You indicated that students are included in this study.

1 * Describe the types of students that are included in this study:

This study has no economic/educational impact on the subject. There is no cost to participate in the study and their is no patient compensation. The patient's standard of care will remain available and unchanged regardless of participation. For these reasons there will be no economic/educational influence or coercion. The consent process will be documented for all patients. The patient's understanding of the study will be confirmed via the appropriateness of their questions and the consent conversation.

2 * Describe how you will prevent undue influence.

In the event that a student is eligible for the study they will be approached about the study. It will be stated that their choice regarding study participation will not impact their employment or position at the institution. Consent will proceed as described in the consent section.

ID: VIEW4E0E519F32000
Name: v2_Vulnerable Populations - Students

Vulnerable Populations - Economically/Educationally Disadvantaged

You indicated that economically or educationally disadvantaged persons are included in this study.

1 * Describe how you will prevent undue influence or coercion with this population.

This study has no economic/educational impact on the subject. There is no cost to participate in the study and their is no patient compensation. The patient's standard of care will remain available and unchanged regardless of participation. For these reasons there will be no economic/educational influence or coercion. The consent process will be documented for all patients. The patient's understanding of the study will be confirmed via the appropriateness of their questions and the consent conversation.

2 * Describe the additional safeguards that have been included in the study to protect the rights and welfare of these participants.

The standard of care for all study patients will remain unchanged. The consent discussion will cover the cost of the study (no cost) and patient compensation (none). The consent process will be documented and will include confirmation of the patient's understanding of the study.

ID: VIEW8D68FCA56796D9B
Name: v2_Vulnerable Populations - Economically/Educationally Disadvantaged

Eligibility

1 * Do you have an existing Eligibility checklist(s) for this study?

◯ Yes ● **No**

1.1 If Yes, upload here. If you need a template, you can download it by clicking **HERE**. The checklists you upload will also be available under the Documents tab of this application.

Name	Created	Modified Date
There are no items to display		

1.2 If No, create an eligibility checklist below:

List inclusion criteria (List each Inclusion Criteria individually, using the ADD button):

	Number	Criteria
View	1	18 years of age or older
View	2	Laboratory-confirmed COVID-19+ by RT-PCR
View	3	Moderate to Severe COVID-19 patients according to FDA's COVID-19 treatment guideline on Management of Persons with COVID-19: Moderate illness is defined as individuals who have evidence of lower respiratory disease by clinical assessment or imaging and a saturation of oxygen (SpO2) >93% on room air at sea level. Severe Illness is defined as individuals who have respiratory frequency >30 breaths per minute, SpO2 ≤ 93% on room air at sea level, ratio of arterial partial pressure of oxygen to fraction of inspired oxygen (PaO2/FiO2) <300, or lung infiltrates >50%.
View	4	Written informed consent must be obtained from patient or LAR (Legally Authorized Representative) before any study procedure is performed

List exclusion criteria (List each Exclusion Criteria individually, using the ADD button):

	Number	Criteria
View	1	Pregnant or breastfeeding women
View	2	Subject has signs or symptoms of acute myocardial ischemia or has required a cardiac intervention within the past 90 days.
View	3	Subject with chronic cardiac conditions including non-vascularized coronary artery disease, heart failure, valvular disease, and cardiomyopathy.
View	4	Subject has an acute or chronic cardiac arrhythmia such as Sinoatrial (SA) or Atrioventricular (AV) Nodal Block/dysfunction, bradycardia, a permanent pace maker, an internal defibrillator, or Atrial Fibrillation/Atrial Flutter requiring treatment or observation.
View	5	Subject has history of Hypotension (sustained systolic blood pressure < 80 mmHg)
View	6	Subject has a history of severe hypertension not adequately controlled with anti-hypertensive medications (Systolic blood pressure ≥ 200 mmHg and/or Diastolic blood pressure ≥ 110 mmHg)
View	7	Subject has moderate or severe renal impairment as well as subject with end stage renal disease (defined as GFR < 60 mL/min/1.73 m2)
View	8	Subject has a history of clinically overt stroke within the past 3 years
View	9	Subject with a history of seizure disorder
View	10	Subject has pre-existing respiratory conditions, most notably asthma or chronic obstructive pulmonary disease or emphysema.
View	11	Subject with respiratory failure for greater than 72 hours. Defined as the continuous use of mechanical ventilation, HFNC >20L/min, CPAP, and/or ECMO. (CPAP use due to obstructive sleep apnea is acceptable).
View	12	Subject who is being treated with chronic anti-coagulation or anti-platelet therapy (prophylactic aspirin is acceptable)
View	13	Subject who is receiving or has received within 30 days any other investigational agents as part of a research study.
View	14	Subject who has received theophylline or aminophylline within 12 hours of study dosing
View	15	Subjects who are currently taking or have taken Persantine and/or Aggrenox within 5 days
View	16	Subjects who have any other clinical conditions that in the opinion of the investigator would make the subject unsuitable for the study.

After entering the inclusion and exclusion criteria above, click the Save link. CICERO will automatically generate a printable Eligibility Checklist for you to use in your research. To review the checklist, click on the resulting link below. This checklist is also available under the Documents tab of this application.

Eligibility Checklist for HP-00091372 v3-3-2021-1614803146921(0.01)

ID: VIEW4E0E5185F9000
Name: v2_Eligibility

Recruitment

1 * Describe plans for recruitment, including the identification of potential participants (or acquisition of charts/records/samples) and initial interactions with them: (If this study involves the VA please list all sites at which recruitment will take place.):

Study staff will screen COVID 19 positive patients via medical records and the COVID Huddle call to determine if they are eligible for the study. Study staff will follow the current institutional plan in regards to competing research studies and enrollment (COVID Huddle call). Study staff will express interest in eligible patients during the COVID huddle call. Due to the nature of the condition and the COVID 19 pandemic, preferred communication methods may change. Patients will be hospitalized and will likely be approached according to the unit policy (video chat etc). LAR's may be approached in person if available or via phone.

Copied from pg. 24 of IND version 1.26.2021
All COVID-19 clinical research studies at the medical center are overseen by the institution's COVID-19 task force committee that helps organize the screening of COVID-19 patients for study enrollment. They have instituted a daily research huddle to facilitate this process and direct potential study subjects to the most appropriate clinical trials. All active studies are invited to this meeting where the newly admitted/diagnosed COVID-19 patients are discussed and presented for consideration of enrollment in clinical trials. This group of clinicians and investigators has been conducting this meeting since Spring 2020 and thus far it has been a very successful mechanism by which patients are enrolled into clinical trials in a fair and clinically relevant approach. We will have a representative from our study team attend this meeting each day and do not anticipate any barriers or conflicts related to competing for clinical trials.

2 * Describe measures that will be implemented to avoid participant coercion or undue influence (if not applicable to the study, enter "N/A"):

We will let patients and family members know this study is voluntary, and participation is not required, and that if they choose not to participate, their care will not be effected.

3 * Who will recruit participants (or acquire charts/records/samples) for this study? (Check all that apply)

- [x] **PI**
- [x] **Study Staff**
- [ ] Third Party

3.1 If you are using a third party, specify Third Party Recruiters:

4 Upload any recruitment tools such as screening/telephone scripts and introductory letters (do not upload advertisements here):

Name	Created	Modified Date
There are no items to display		

ID: VIEW4E0BCAA0A6C00
Name: v2_Recruitment

Advertising

1 * Will you be using advertisements to recruit potential participants?

○ Yes ● **No**

ID: VIEW4E0BCCF811000
Name: v2_Advertising

Research Related Risks

If you uploaded a separate research protocol document in the 'Research Protocol' page, cite the applicable section and page numbers from that document in the answer box below.

1 * Individually list each research-related risk, using a separate line for each. Next to each risk, delineate the likelihood/seriousness of the risk, and the provisions for minimizing the risk:

Risks and side effects related to the regadenoson infusion include:

Likely
• Headache
• Flushing
• Chest discomfort
• Shortness of breath
• Dizziness
• Chest pain (angina)
• Nausea and/or vomiting
• Abdominal pain
• Muscle pain

Less Likely
• Changes in blood pressure
• Heart rhythm changes
• Allergic reaction

Rare but serious
• Heart attack which could cause death
• Seizure
• Stroke

Aminophylline, a competitive adenosine receptor antagonist, will be available during the infusion as a rescue medication for rare side effects of Regadenoson, such as bronchoconstriction. Aminophylline has been used to terminate persistent pharmacodynamic effects. In such case, aminophylline may be administered in doses ranging from 50 mg to 250 mg by slow intravenous injection (50 mg to 100 mg over 30–60 seconds).

Medicines such as caffeine, theophylline or aminophylline can interfere with Regadenoson. Recent use of these medications is an exclusion for the study.

Blood draw risks:
• pain,
• a bruise,
• fainting or passing out, and
• infection

Risks for women:
Unknown risks to pregnany woman. Pregnancy is an exclusion.

Loss of confidentiality will be minimized by storing data in a secure location such as a locked office and locked cabinet and electronic data will be password-protected.

Potential Benefits and Alternatives

If you uploaded a separate research protocol document in the 'Research Protocol' page, cite the applicable section and page numbers from that document in the answer boxes below.

1 * Describe the potential direct benefit(s) to participants:

There may or may not be a direct benefit to participating in this trial.

Possible benefits include: The moderate COVID-19 patients may prevent to be on intubation or ventilator.
The severe COVID-19 patients may increase survival
The length of hospital stay and ICU stay may be shorter.
The COVID 19 symptoms may be resolved quicker.

2 * Describe the importance of the knowledge expected to result from the study:

Based on our previous results with A2AR agonists in acute lung injury and lung transplantation we anticipate that regadenoson will: 1) be safe and well tolerated by COVID-19 patients; 2) reduce lung hyperinflammation; 3) enhance lung function (measured by pulse oximetry, ventilator parameters, arterial blood gases and chest X-ray), 4) reduce levels of circulating pro-inflammatory cytokines and enzymes; and 5) increase survival on day 30 of COVID-19 patients by 10% or more.

3 * Describe how the potential risks to participants are reasonable in relationship to the potential benefits:

The risks are side effects of the Infusion of regadenson, but should be minimal based on our data already obtained in our ongoing phase I trial in lung transplantation patients.
The benefit is potential effective treatment for COVID-19. which include:
The moderate COVID-19 patients may prevent to be on intubation or ventilator.
The severe COVID-19 patients may increase survival
The length of hospital stay and ICU stay may be shorter.
The COVID-19 symptoms may be resolved quicker.
Considering both the potential risks and the benefits, it is reasonable to have this trial.

4 * Describe the alternatives to participation in this study. If there are no alternatives, state that participation is voluntary and the alternative is not to participate. For intervention studies, describe appropriate alternative clinical procedures or courses of treatment available to subjects.

This study is voluntary and the alternative is to not participate. Additionally the participant may be eligible for other study treatments. The participant's decision to participate will have no impact on their future healthcare.

ID: VIEW4E1B5251B0400
Name: v2_Potential Benefits and Alternatives

Withdrawal of Participants

If the questions below are not applicable to the research (i.e., chart review), enter "N/A".

1 * Describe anticipated circumstances under which subjects will be withdrawn from the research without their agreement:

Dr. Christine Lau can remove patients from the study at any time. Some of the reasons for doing so may include
a) concern for patient clinical status
b) Worsening clinical status
c) Severe side effects
d) New information shows the treatment will not work or is not safe
e) Patient failure to follow instructions
f) The study sponsor closes the study for safety, administrative or other reasons

2 * Describe procedures for orderly termination:

Patients are asked to notify the PI or study staff. They will sign a withdraw form if willing. The form can be found under additional documents.

3 * Describe procedures that will be followed when subjects withdraw from the research, including partial withdrawal from procedures with continued data collection:

If a subject withdraws, the data already collected will not be destroyed. Further data will not be collected. Partial withdrawal may occur and subject data will continue to be collected.

ID: VIEW4E1B52531F800
Name: v2_Withdrawal of Participants

Privacy of Participants

If the study does not involve interaction with participants, answer "N/A" to the questions below.

1 * Describe how you will ensure the privacy of potential participants throughout the study ***(privacy refers to persons and their interest in controlling access to themselves):***
All study participants will be evaluated and examined for study-related activities in a private room. Every possible effort will be made to maintain the privacy and confidentiality of the participant's name and other identifying information. The process of consent will be performed in the doctor's office or the hospital, which is a HIPAA compliant area. All the participant's files will be stored with proper locks and keys. All the paper files are secured in cabinets with locks and keys that are always in a secure place and only the PI and research staff have access to these cabinets.

2 * Describe the location where potential participants will receive research information and detail the specific actions the study team will take to ensure adequate privacy areas:
COVID-19 patients are isolated in single, private hospital rooms. The clinical staff can take the consent form into the isolation area and give it to the patient to review. The PI and/or study team will discuss the consent via phone or videochat. Alternatively, the consent form can be sent via iPad if available. The consent will still be reviewed over the phone with all parties in private locations.

If discussing with a patient's LAR, the consent may be discussed in person if possible or it will be electronically sent and reviewed with the LAR over the phone.

The study team will have these discussions in private locations to maintain patient privacy.

3 * Describe potential environmental stressors that may be associated with the research:
COVID-19 is a stressor in and of itself. We will try to limit that stress by talking to patients/LARs at an appropriate time (not rushed or when other people are trying to talk about other items).

4 * Will this study have a site based in the European Union?
○ Yes ● **No**

5 * Will the study have planned recruitment or data collection from participants while they are located in the European Union?
○ Yes ● **No**

Access link below for information about the EU General Data Protection Regulations to assist in answering these questions.
https://www.umaryland.edu/oac/general-data-protection-regulation/

Confidentiality of Data

1 * Will stored research data contain identifiers or be able to be linked to and identify individual participants (either directly or through a code/research ID)?

- (●) **Yes**
- ( ) No, the data will be stored de-identified/anonymous (stripped of all identifiers, no way to identify individual participants)

2 * Where will research data be kept (address electronic and paper data as applicable)? (If this is a VA study please list specific sites that data will be kept.)

Data may be kept in the clinical research office at 110 S. Paca St in the cardiac surgery research office. All electronic data will be kept on password protected computers.

3 * How will such data be secured?

All data is kept password protected computers and in locked offices and buildings.

4 * Who will have access to research data?

PI and study staff

5 * Will study data or test results be recorded in the participant’s medical records?

( ) Yes (●) **No**

6 * Will any data be destroyed? ***(Please note that data for FDA regulated research and VA research cannot be deleted)***

( ) Yes (●) **No**

6.1 If Yes, what data (e.g., all data, some recordings, interview notes), when and how?

7 Do you plan to obtain a Certificate of Confidentiality?

( ) Yes (●) **No**

7.1 If Yes, upload your Certificate of Confidentiality. If you have not yet obtained the Certificate, please note that once it is obtained, you will need to submit an amendment to attach the document, make any needed changes to the submission and make needed changes to the Informed Consent Document.

Name	Created	Modified Date
There are no items to display		

8 * Discuss any other potential confidentiality issues related to this study:

NA

Monitoring Plan Selection

1 * Type of data safety monitoring plan for the study:

- ○ Will use/defer to the external sponsor's Data Safety Monitoring Plan
- ● **Data Safety Monitoring by a Committee**
- ○ Data Safety Monitoring by an Individual
- ○ There is no data safety monitoring plan in place

ID: VIEW4E1B00E30D400
Name: v2_Monitoring Plan Selection

Monitoring Plan - Committee

You indicated that the monitoring will be done by a Committee.

1 * Will the Committee be Internal or External?

- ○ Internal DSMB
- ● **External DSMB**

2 * What data will be reviewed?

- [x] **Adverse Events**
- [x] **Enrollment Numbers**
- [ ] Patient Charts/Clinical Summaries
- [ ] Laboratory Tests
- [x] **Medical Compliance**
- [ ] Procedure Reports
- [ ] Raw Data
- [x] **Outcomes (Primary, Secondary)**
- [ ] Preliminary Analyses
- [ ] Other

2.1 If Other, specify:

3 * What will be the frequency of the review?

- ○ Annually
- ○ Bi-Annually
- ● **Other**

3.1 If Other, specify:
Meetings will be scheduled based on enrollment. They will occur after the completion of cohort 1 as described in the safety assessment section. Meetings will also be scheduled after enrollment (defined as start of study infusion) of 15, 25, and 40 subjects, with additional meetings or conference calls scheduled as needed to review SAEs or other issues.
Please see pg. 26 of IND version 1.26.2021

4 * Safety monitoring results will be reported to:

- [x] **IRB**
- [ ] GCRC
- [x] **Sponsor**
- [ ] Other

4.1 If Other, specify:

Research-Related Costs

1 * Is the study's financial supporter (e.g., commercial sponsor, federal or state grant or contract, private foundation, physician-sponsor) covering any research-related costs?

- ○ No
- ● **Yes**

1.1 If Yes, check all that apply:

- [x] **Research-Related Services (personnel costs, tests, supplies, exams, x-rays, or consultations required in the study)**
- [ ] Investigational or Study Device
- [x] **Investigational or Study Drug**
- [ ] Investigational Procedure(s)

1.2 If No, who is responsible for payment?

2 * Who is responsible for the uncovered research-related costs?

- [ ] Participant
- [ ] Sponsor
- [ ] UM
- [ ] Other
- [x] **There will be no uncovered research-related costs**

2.1 If Other, specify:

3 If the participant is responsible for any research-related costs, identify and estimate the dollar amount:

Compensation for Research-Related Injury

1 * Is this study under a master agreement that includes a provision requiring the sponsor to provide compensation to participants for research-related injury?

○ Yes ● **No**

1.1 If Yes, please provide the date and title of the agreement and upload the portion of the contract language relevant to compensation for research-related injury:

Name	Created	Modified Date
There are no items to display		

1.2 If No (the study is not under a master agreement), is there proposed contract language concerning payment to participants for treatment in the event of a research-related injury?

○ Yes ● **No**

1.2.1 If Yes, indicate the status of the contract review/approval with the ORD and upload the proposed language relevant to compensation for research-related injury:

1.2.2

Name	Created	Modified Date
There are no items to display		

ID: VIEW4E1B629EEC000
Name: v2_Compensation for Research-Related Injury

Payment/Reimbursement to Participants

1 * Will participants receive payment (money, gift certificates, coupons, etc.) or reimbursement for their participation in this research?

○ Yes ● **No**

HIPAA (Health Insurance Portability and Accountability Act)

1 * Are you affiliated with, or will you be accessing data from a HIPAA-covered entity? A covered entity might be a hospital, a physician practice, or any other provider who transmits health information in electronic form.

• At UMB, this includes UMB schools designated as covered entities (School of Medicine and School of Dentistry) and entities under the University of Maryland Medical System (UMMS). The Baltimore VA Medical Center is also a covered entity.

• If you are a researcher from any school that is not a covered entity but is accessing electronic medical records from a covered entity (such as UMMC), HIPAA would be applicable. Please see a list of covered entities included under UMMS here: *executed-ace-designation-042018.pdf*

(●) **Yes** ( ) No

2 * If Yes, will the study view, access, share, collect, use, or analyze health information that is individually identifiable under HIPAA?

(●) **Yes** ( ) No

Protected Health Information (PHI)

You indicated that HIPAA applies and the study will view, access, share, collect, use, or analyze health information that is individually identifiable.

1 * Which PHI elements will be used or disclosed in this study? (Check all that apply)

- [x] **Name**
- [ ] Address (if more specific than Zip Code)
- [x] **Dates**
- [x] **Ages over age 89**
- [x] **Telephone numbers**
- [ ] Fax numbers
- [x] **Email addresses**
- [ ] Social Security numbers
- [x] **Medical record numbers**
- [ ] Health plan beneficiary numbers
- [ ] Account numbers
- [ ] Certificate/license numbers
- [ ] Vehicle identifiers and serial numbers, including license plate numbers
- [ ] Device identifiers and serial numbers
- [ ] Web universal resource locators (URLs)
- [ ] Internet protocol (IP) address numbers
- [ ] Biometric identifiers, including fingerprints and voiceprints
- [ ] Full-face photographic images and any comparable images
- [ ] Any other unique identifying number, characteristic, or code, unless otherwise permitted by the Privacy Rule for re-identification
- [ ] None

2 * Why is the PHI necessary for this research?
If SSNs are going to be used, describe the specific use and type of SSN to be used (real, scrambled, last 4 digits).
Medical record numbers are needed because we need to be able to monitor our study patients over specified period of time. In order to contact patients, we also need phone numbers or e-mail address for consenting and the 30-day follow-up.

3 * What is the source(s) of the PHI?
Medical records

4 * Provide written assurance that Protected Health Information will not be reused. (Note: this refers to re-use on another study or for a purpose which has not been approved, not to the re-use of screening data during the current study).
No PHI will be reused.

5 * How will permission to allow the use/disclosure of the individual's protected health information (PHI) be obtained? (Choose all that apply:)

- [x] **Obtain written authorization** ***(upload authorization form at the end of the application under "Consent and HIPAA Authorization Forms")***
- [x] **Requesting waiver/alteration of authorization (includes waiver of authorization for recruitment only)**
- [ ] Qualifies as a limited data set (LDS)

5.1 If you are using a limited data set (LDS), please attach the Data Use Agreement (DUA):

Name	Created	Modified Date

There are no items to display

Waiver/Alteration of Authorization

You indicated that a waiver/alteration of authorization is requested.

1 * Provide rationale for how the research presents no more than minimal risk to the privacy of individuals:

A waiver is requested for the screening purpose only. We will obtain written consent for study participation. The medical records used for screening purposes are obtained mainly from the hospital information system (EPIC), and this database meets regulatory requirements as well as CMS requirements. Therefore the use of this database to screen patients' eligibility will not pose any additional risks compared to non-study participants. Furthermore, data will only be viewed for eligibility purposes. Data will not be stored if participants are found to be ineligible or if they decline to participate.

2 * Describe the plan to ensure the protection of PHI collected during this study from improper use and disclosure:

As explained in #1, the hospital information systems are secured, and are compliant. People who work at the UMMC already have access to these systems, however, research staff members do not have any editing rights. The information used to screen patients will not be shared with people outside our department. Additionally, if we need to print out any information, it will be kept in our office (locked when no one is present).

3 * Describe the plan to destroy the PHI collected during this study at the earliest opportunity consistent with the conduct of the research. If there is a need to retain PHI, provide a justification:

If a patient consents to participate, we will retain the information until sponsor-required retention period ends, and it will be destroyed according to a sponsor's instruction. If a patient does not meet eligibility criteria or does not consent, all information printed will be destroyed in the same manner as other medical records used for non-research purposes.

4 * Why could the research not practicably be done without access to and use of this PHI?

We cannot assess any one's eligibility without medical records.

5 * Why could the research not practicably be done without the waiver or alteration?

We cannot assess any one's eligibility without medical records

6 * Will the subjects' PHI be disclosed to (or shared with) any individuals or entities outside of UM?

○ Yes ● **No**

6.1 If Yes, describe the individuals or entities outside of UM to whom PHI will be disclosed.

Informed Consent Process

If the study does not involve interaction with participants or a waiver of consent is being requested , answer "N/A" to the questions below.

1 * Indicate the type(s) of consent that will be involved in this study: (check all that apply)

- [ ] Not applicable (study may qualify as exempt)
- [ ] Request to Waive Consent/Parental Permission (Consent is not being obtained)
- [ ] Request to Alter Consent (Some Elements of Consent Waived)
- [ ] Request to Waive Documentation of Consent (Verbal/Oral Consent)
- [x] **Written Consent Form**
- [ ] Electronic Consent

2 * Describe the Informed Consent process in detail:

Consenting COVID-19 patients is very difficult. We will absolutely adhere to the guidelines provided by HRPO.

If a patient is awake and alert, and in a containment unit or quarantined space; A photograph of the signed ICF can be transmitted to trial staff. An unsigned ICF is provided to the patient by a clinical staff member who has entered the room. The PI or research team member arranges a telephone call, videoconference call, or a Zoom call with the patient. The ICF will be reviewed with the patient by the PI or research team member, who will respond to any questions the patient may have. Verbal confirmation by the patient that their questions have been answered that they would like to participate in the trial and that they have signed and dated the ICF that is in their possession.

The patient (or the clinical staff member in the room) takes a photograph of the signed ICF and sends it to the PI or research team member. The PI or research team member signs a printed copy of the ICF photograph and stores the signed ICF in the study pts records/ binder along with a consent note that states the complete process.

If the patient is intubated and/or cognitively impaired the study will be discussed with the patient's LAR. A written consent form will be completed in person if the LAR is local and visitor policies allow them to be present. The evaluation of consent worksheet will be used and can be found in the vulnerable subjects - cognitively impaired section.

In the event that it is not feasible for the LAR to be present or restrictions prevent their presence then the written consent process will occur via email/mail. The LAR will be initially contacted via phone and an email address will be requested. The ICF will be emailed to the LAR for their review. Alternatively, the consent may be mailed. Upon receipt of the consent, the PI or designated study member will discuss all elements of the consent and answer any questions. The LAR will be given time to review the consent and discuss with family members as needed. If the LAR agrees to participation then they will print the document if needed and sign it. If the LAR has the capability they may alternatively sign via a verified electronic signature that includes the date and time. Preferably the document will be scanned and returned via email. The document may also be photographed and sent via message/email. The original copy signed by the LAR will be mailed to the study staff if other options are not feasible. A copy of the document signed by the LAR will be signed by the PI/designated study member and kept in the research files. A copy with all required signatures will be emailed or mailed to the LAR.

3 * Confirm that the consent process will explain the following:

- The activities involve research.
- The procedures to be performed.
- That participation is voluntary.
- The name and contact information for the investigator.

(●) **Yes** ( ) No

4 * Describe who will obtain Informed Consent:

The PI and a member of the study team

5 * If obtaining consent from a legally authorized representative (LAR), describe how you will confirm that the individual is the LAR and can provide legally effective informed consent. *(Answer "N/A" if not obtaining consent from LARs)*

We will discuss with the nursing team taking care of the patients in the COVID-19 rooms. We will also confirm via medical records. The LAR identification form will be used. This can be found under additional documents.

6 * Describe the setting for consent:

Settings can be in multiple places. The patient may be approached via phone or videochat/ipad while they are in the COVID-19 suites. LAR consent may occur in a number of settings due to the restricted visitor policy and nature of COVID-19 restrictions.

7 * Describe the provisions for assessing participant understanding:

Please see attached "eval to sign consent" in vulnerable subjects - cognitively impaired section.

8 * Describe the consideration for ongoing consent:

In the case of an LAR signing the initial consent, the patient's cognitive status will be monitored. If and when the patient becomes cognitively unimpaired they will be reconsented in a timely fashion. If the participant has passed the study end at 30 days post infusion then reconsent will not be required.
Outside this circumstance we do not anticipate that participants will have to re-consent during their participation.

Non-English Speakers

You indicated that participants may speak languages other than English.

1 * Describe how you will explain the study and ensure that the non-English speaking subjects understand the study and their participation in research:
A certified interpreter will be utilized to complete the consent discussion. All aspects of the consent discussion will be completed including answering any questions that the patient may have. The evaluation to sign consent form will be completed.

2 * Indicate the method of translation:

- (●) **Investigator will provide IRB with translation of approved consent form**
- ( ) Translated short form consent form will be used with an interpreter along with a study summary document (Limited Use)

3 If the research will primarily include subjects who speak a language other than English, the informed consent documents should be translated into that language. Indicate the language(s) and method of translation.
Consent forms have been translated into Spanish with the certificate of translation from an accredited agency.

Upload the certificate of translation:

Name	Created	Modified Date
Translation Certificate(0.01)	4/29/2021 10:18 AM	4/29/2021 10:18 AM

4 * Indicate whether or not an interpreter will be used. If so, how will you guarantee that the interpreter will maintain confidentiality of subjects? For whom does the interpreter work and how will the interpreter be recruited for the study?
A Spanish interpreter will be used through HIPAA compliant agency used by our institution. The interpreter will be requested via phone or Ipad based on which is available.

5 * Indicate who will be responsible for updating subjects about study progress or any changes, collecting complaints, etc. during the course of the study:
The study team with the assistance of an interpreter.

6 If this research will be conducted outside the United States, indicate subjects' native language and literacy level:
n/a

ID: VIEW4E1B2E3D38400
Name: v2_Non-English Speakers

Consent and HIPAA Authorization Forms - Draft

1 Upload all of your Consent Forms for approval. Use only Microsoft Word.

Name	Created	Modified Date
06-HP-00091372 COVID-19 Non-Randomized_3.3.21_SPANISH(0.01)	4/29/2021 10:22 AM	4/29/2021 10:22 AM
HP-00091372 COVID-19 Randomized_3.3.21_SPANISH(0.01)	4/29/2021 10:23 AM	4/29/2021 10:23 AM
06-HP-00091372 COVID-19 Non-Randomized_3.3.21_clean.docx(0.01)	3/3/2021 4:30 PM	3/3/2021 4:30 PM
HP-00091372 COVID-19 Randomized_3.3.21_clean.docx(0.01)	3/3/2021 4:30 PM	3/3/2021 4:30 PM

IMPORTANT NOTE: the above list of consent forms (if any) are DRAFT versions. Under no circumstances should copies of these be distributed to patients/study subjects. If/when this research submission is approved by the IRB, approved consent forms will be available for download and use from the "Documents" tab of the Submission's workspace (click Exit and then look for the Documents tab - approved submissions only)

1A Archived Consent Forms:

Name	Created	Modified Date
HP-00091372 COVID-19 Randomized_1.26.2021_clean.docx(0.01)	2/10/2021 2:05 PM	2/10/2021 2:05 PM
06-HP-00091372 COVID-19 Non-Randomized_1.26.2021_ clean.docx(0.01)	2/10/2021 2:05 PM	2/10/2021 2:05 PM
ICF v1 HP-00091372 COVID-19 Open Enrollment_10.1.20.docx(0.01)	10/1/2020 10:36 AM	10/1/2020 10:36 AM
ICF v1 HP-00091372 COVID-19 Study Randomized_10.1.20.docx(0.01)	10/1/2020 10:35 AM	10/1/2020 10:35 AM
ICF v1 HP-00091372 COVID-19 Study.docx(0.01)	8/28/2020 3:59 PM	8/28/2020 3:59 PM

2 Upload any HIPAA authorization forms here:
There are no items to display

Please refer to HRPO's website for specific instructions for preparing informed consent documents and to access current templates:
http://hrpo.umaryland.edu/researchers/consents.html

Organization Review Requirements (other than IRB)

Answer the following questions to determine additional organizational review requirements:

1 **Department/Division Review** - All research submissions are required to undergo department/division/institutional review prior to IRB review. The following entity is listed as the required department/division/institutional review:

Surgery

If this information is incorrect, please notify the HRPO office.

2 **RSC Review Criteria** - select 'Yes' if the answer is 'Yes' for any of the following questions. Review by the Radiation Safety Committee may be required.

* 2.1 Does the research involve the use of ionizing radiation? ○ Yes ● **No**

2.2 Does the research involve the sampling of radioactive human materials for subsequent use or analysis in a laboratory?

3 **IBC Review Criteria** - select 'Yes' if the answer is 'Yes' for any of the following questions. Review by the Institutional Biosafety Committee may be required.

* 3.1 Does the research involve human gene transfer? ● **Yes** ○ No
-OR-
Does the research specifically apply to human studies in which induction or enhancement of an immune response to a vector-encoded microbial immunogen is the major goal, and such an immune response has been demonstrated in model systems, and the persistence of the vector-encoded immunogen is not expected? This type of research is often referred to as recombinant vaccine trials.

3.2 Does the research involve the exposure of human subjects to pathogenic microorganisms, or the exposure of research staff to human subjects or samples known or reasonably expected to carry infectious disease(s)?

3.3 Does the research involve the sampling of materials from persons with no known infectious disease and where the only risk to study staff is occupational exposure to bloodborne pathogens as defined by the OSHA Bloodborne Pathogen Standard?

4 **Cancer Center Criteria** - Answer the following to determine if review by the Cancer Center (Hematology-Oncology) may be required.

* Does the protocol involve in any way studies related to the prevention, treatment, diagnosis, or imaging of neoplastic diseases? ○ Yes ● **No**

5 **General Clinical Research Center Review Criteria** - the GCRC offers free and/or cost shared resources for patient-oriented research. Click Here for more information.

Answer the following to determine if review by the GCRC may be required.

* Will the General Clinical Research Center (GCRC) facility or resources be used to conduct this activity? ○ Yes ● **No**

6 **VA Review Criteria** - Answer the following questions to determine if review by the VAMHCS R&D Committee may be required.

* 6.1 - Will the research be conducted by VA Investigators including PIs, Co-PIs, and Site Investigators on VA time (serving on compensated, WOC, or IPA appointments)? ○ Yes ● **No**

* 6.2 - Will the research utilize VA resources (e.g., equipment, funds, medical records, databases, tissues, etc.)? ○ Yes ● **No**

* 6.3 - Will the research be conducted on VA property, including space leased to and used by VA? ○ Yes ● **No**

PLEASE NOTE that the research may be funded by VA, by other sponsors, or may be unfunded.

Institutional Biosafety Committee Review Required

1 **NOTE**: based on your answers to questions on a previous page (see below) review by the Institutional Biosafety Committee (IBC) is required. This will involve extra steps on your (study team) part. Clicking the Continue button will result in the system creating a blank IBC Submission form for you. You will be required to fill out and submit this IBC form before you will be able to submit the Protocol form. The IBC Submission workspace and form can be reached by clicking the appropriate button on the left hand side of the Protocol submission's workspace (web page) after exiting the Protocol form.

2 **Question** - answered on IBC RSC review requirements page:

3.1 Does the research involve human gene transfer? - OR - Does the research specifically apply to human studies in which induction or enhancement of an immune response to a vector-encoded microbial immunogen is the major goal, and such an immune response has been demonstrated in model systems, and the persistence of the vector-encoded immunogen is not expected? This type of research is often referred to as recombinant vaccine trials.
3.2 Does the research involve: a) the exposure of human subjects to pathogenic microorganisms, or b) the potential exposure of UMB research staff to infectious materials through the sampling or processing of materials from patients with known infectious disease or from environmental surfaces?
3.3 Does the research involve the sampling of materials from persons with no known infectious disease and where the only risk to study staff is occupational exposure to bloodborne pathogens as defined by the OSHA Bloodborne Pathogen Standard?

Yes

If the answer to this question is wrong, an IBC submission is not required, use the Jump To menu or your browser's <

3 * **Confirm** - you have read the above information and understand that in addition to the IRB Protocol form, you will fill out and submit the IBC Submission form :

● **Yes** ○ No

ID: VIEW4E1AF91ED4C00
Name: v2_Institutional Biosafety Committee Review Required

Summary of Required Reviews (other than IRB)

1 **Additional Committee Reviews** - Based on your responses to the previous questions, you have identified the following additional reviews. To complete or view these additional committees' forms, click on the links below or exit this application and click on the appropriate button on left side of this submission's webpage.

Name of Related Submission		
IBC: Regadenoson COVID-19 Trial (HP-00091372)	Workspace	SmartForm

2 **Required Department and Specialty Reviews** - Based on the PI's organization (department, division, etc.) affiliation and answers to previous questions (use of Cancer Center, etc.), the organizations listed below are required to review this application. These reviews are conducted online and no additional forms or steps by the study team are required.

Name of Organization	**Review Status**
Surgery	Complete
SOM Program in Trauma	Pending

ID: VIEW4E1C8D9AE4000
Name: v2_Summary of Required Reviews (other than IRB)

Additional Documents

1

Upload all additional documents here:

Name	Created	Modified Date
HP-00091372 COVID-19 Randomized_3.3.21_tracked.docx(0.01)	3/3/2021 4:30 PM	3/3/2021 4:30 PM
06-HP-00091372 COVID-19 Non-Randomized_3.3.21_tracked.docx(0.01)	3/3/2021 4:30 PM	3/3/2021 4:30 PM
Task Force Response Letter Signed.doc(0.01)	2/12/2021 4:59 PM	2/12/2021 4:59 PM
IRB Response Letter Signed.doc(0.01)	2/12/2021 4:59 PM	2/12/2021 4:59 PM
Lung Tx DSMB IRB Memo February 2021- DSMB APPROVAL.pdf(0.01)	2/10/2021 2:06 PM	2/10/2021 2:06 PM
Eval to Sign Consent Form.pdf(0.01)	12/2/2020 12:52 PM	12/2/2020 12:52 PM
IND 149635 Study May Proceed (COR-INDAD-01).pdf(0.01)	10/14/2020 10:39 AM	10/14/2020 10:39 AM
Detailed DSMB plan_10.9.20.docx(0.01)	10/9/2020 11:27 AM	10/9/2020 11:27 AM
UMB COVID Risk Statement 09.2020 FINAL_91372.docx(0.01)	10/1/2020 9:38 AM	10/1/2020 9:38 AM
Clinical-Research-Resumption-Plan---Proposed-Assessment_HP-91372.pdf(0.01)	9/17/2020 12:47 PM	9/17/2020 12:47 PM
Clinical-Research-Resumption-Checklist_HP-91372.pdf(0.01)	9/17/2020 12:46 PM	9/17/2020 12:46 PM
LAR Identification Form.pdf(0.01)	9/16/2020 5:39 PM	9/16/2020 5:39 PM
withdrawl form(0.01)	5/6/2020 3:34 PM	5/6/2020 3:34 PM

ID: VIEW4E0962513A000
Name: v2_Additional Documents

Final Page of Application

You have reached the final page of this application. It is recommended that you click on the "Hide/Show Errors" link on the upper or lower breadcrumb row of this page. The "Hide/Show Errors" will do a search of your application, and highlight areas that are required or need to be completed prior to submitting.

By submitting this application, you are electronically routing the protocol for departmental scientific review and all other necessary reviews. According to information you have provided, this application will be routed to the following Departments for review prior to being forwarded to the IRB for review. These reviews are conducted online and no additional forms or steps by the study team are required.

Name of Organization	**Review Status**
Surgery	Complete
SOM Program in Trauma	Pending

Required Safety Committee Reviews - In addition to the IRB, the following committees must review this submission. Each additional committee has a separate online form that the study team will be required to fill out. All committee applications (IRB plus those listed here) must be completed properly before the 'package' of applications can be submitted. The team may complete these additional forms in any order or at any time prior to submission of the IRB Application. To complete or view these additional committees' forms, click on the links below or exit this application and click on the appropriate button on left side of this submission's Workspace.

Name of Related Submission		
IBC: Regadenoson COVID-19 Trial (HP-00091372)	Workspace	SmartForm

You may check the progress of your application at any time by returning to the Workspace of this submission. A detailed history, including notes, dates, and times of events, is provided to you for this purpose.

If a reviewer returns the application to you, you must address their concerns and resubmit the protocol for review to all designated departments. After all departments have reviewed the application, it will automatically be sent to the IRB for review. Changes made to the submission after its approval must be submitted as modifications.

Investigator Attestation
By submitting this application, I, the Principal Investigator (PI), certify that the information provided in this application is complete and correct. Research will be conducted according to the submission as described, only by the approved principal investigator and study team members.

In addition, I agree to the responsibilities of a PI, including:

- Obtaining informed consent (if applicable) from all subjects as outlined in the submission.
- Reporting new information to the IRB per the requirements of the Investigator Manual.
- If Required, obtaining renewal of the protocol prior to the expiration of the approval period or halt all study activities upon study expiration.
- Accepting ultimate responsibility for the protection of the rights and welfare of human subjects, conduct of the study and the ethical performance of the project.
- Ensuring performance of all research activities by qualified personnel according to the IRB approved submission.
- Ensuring that research personnel have or will receive appropriate training.
- Ensuring no changes will be made in the research until approved by the IRB (except when necessary to eliminate apparent immediate hazards to subjects).

Click the "Finish" button and then click "Submit Application" in the submission Workspace.

Add a Team Member

1 * Select Team Member:
Freshta Akbari

2 Research Role:
Research Team Member

3 * Edit Rights - Should this person be allowed full edit rights to the submission, including: editing the online forms and the ability to execute activities? Note - a person with edit rights will automatically be added to the CC list and will receive all emails regarding this protocol, even if the answer to #4 below is No.

● **Yes** ○ No

4 * CC on Email Correspondence - Should this person be copied on all emails sent by the HRPO office to the PI/POC? Study team members with edit rights will automatically receive all emails:

● **Yes** ○ No

5 * Does this study team member have a potential conflict of interest, financial or otherwise, related to this research?

○ Yes ● **No**

6 * Briefly describe experience conducting research and knowledge of the local study sites, culture, and society:
Freshta has extensive research experience.

Add a Team Member

1 * Select Team Member:
Matthew Audette

2 Research Role:
Research Team Member

3 * Edit Rights - Should this person be allowed full edit rights to the submission, including: editing the online forms and the ability to execute activities? Note - a person with edit rights will automatically be added to the CC list and will receive all emails regarding this protocol, even if the answer to #4 below is No.
● **Yes** ○ No

4 * CC on Email Correspondence - Should this person be copied on all emails sent by the HRPO office to the PI/POC? Study team members with edit rights will automatically receive all emails:
● **Yes** ○ No

5 * Does this study team member have a potential conflict of interest, financial or otherwise, related to this research?
○ Yes ● **No**

6 * Briefly describe experience conducting research and knowledge of the local study sites, culture, and society:
Matthew has research experience.

Add a Team Member

1 * Select Team Member:
Melissa Culligan

2 Research Role:
Study Coordinator

3 * Edit Rights - Should this person be allowed full edit rights to the submission, including: editing the online forms and the ability to execute activities? Note - a person with edit rights will automatically be added to the CC list and will receive all emails regarding this protocol, even if the answer to #4 below is No.
● **Yes** ○ No

4 * CC on Email Correspondence - Should this person be copied on all emails sent by the HRPO office to the PI/POC? Study team members with edit rights will automatically receive all emails:
● **Yes** ○ No

5 * Does this study team member have a potential conflict of interest, financial or otherwise, related to this research?
○ Yes ● **No**

6 * Briefly describe experience conducting research and knowledge of the local study sites, culture, and society:
research nurse

Add a Team Member

1 * Select Team Member:
Yunge Zhao

2 Research Role:
Research Team Member

3 * Edit Rights - Should this person be allowed full edit rights to the submission, including: editing the online forms and the ability to execute activities? Note - a person with edit rights will automatically be added to the CC list and will receive all emails regarding this protocol, even if the answer to #4 below is No.
(●) **Yes** ( ) No

4 * CC on Email Correspondence - Should this person be copied on all emails sent by the HRPO office to the PI/POC? Study team members with edit rights will automatically receive all emails:
(●) **Yes** ( ) No

5 * Does this study team member have a potential conflict of interest, financial or otherwise, related to this research?
( ) Yes (●) **No**

6 * Briefly describe experience conducting research and knowledge of the local study sites, culture, and society:
PhD who will be processing and analyzing samples, analyzing data and write report

Add a Team Member

1 * Select Team Member:
Manal Al-Suqi

2 Research Role:
Research Team Member

3 * Edit Rights - Should this person be allowed full edit rights to the submission, including: editing the online forms and the ability to execute activities? Note - a person with edit rights will automatically be added to the CC list and will receive all emails regarding this protocol, even if the answer to #4 below is No.
● **Yes** ○ No

4 * CC on Email Correspondence - Should this person be copied on all emails sent by the HRPO office to the PI/POC? Study team members with edit rights will automatically receive all emails:
● **Yes** ○ No

5 * Does this study team member have a potential conflict of interest, financial or otherwise, related to this research?
○ Yes ● **No**

6 * Briefly describe experience conducting research and knowledge of the local study sites, culture, and society:
Manal is a research team member.

Add a Team Member

1 * Select Team Member:
Joseph Rabin

2 Research Role:
Sub-Investigator

3 * Edit Rights - Should this person be allowed full edit rights to the submission, including: editing the online forms and the ability to execute activities? Note - a person with edit rights will automatically be added to the CC list and will receive all emails regarding this protocol, even if the answer to #4 below is No.

○ Yes ● **No**

4 * CC on Email Correspondence - Should this person be copied on all emails sent by the HRPO office to the PI/POC? Study team members with edit rights will automatically receive all emails:

○ Yes ● **No**

5 * Does this study team member have a potential conflict of interest, financial or otherwise, related to this research?

○ Yes ● **No**

6 * Briefly describe experience conducting research and knowledge of the local study sites, culture, and society:
ICU physician familiar with COVID patients

Add a Team Member

1 * Select Team Member:
Ezzat Mostafa

2 Research Role:
Research Team Member

3 * Edit Rights - Should this person be allowed full edit rights to the submission, including: editing the online forms and the ability to execute activities? Note - a person with edit rights will automatically be added to the CC list and will receive all emails regarding this protocol, even if the answer to #4 below is No.

● **Yes** ○ No

4 * CC on Email Correspondence - Should this person be copied on all emails sent by the HRPO office to the PI/POC? Study team members with edit rights will automatically receive all emails:

● **Yes** ○ No

5 * Does this study team member have a potential conflict of interest, financial or otherwise, related to this research?

○ Yes ● **No**

6 * Briefly describe experience conducting research and knowledge of the local study sites, culture, and society:
Ezzat has prior research and clinical experience.

Add a Team Member

1 * Select Team Member:
Alexander Krupnick

2 Research Role:
Sub-Investigator

3 * Edit Rights - Should this person be allowed full edit rights to the submission, including: editing the online forms and the ability to execute activities? Note - a person with edit rights will automatically be added to the CC list and will receive all emails regarding this protocol, even if the answer to #4 below is No.

○ Yes ● **No**

4 * CC on Email Correspondence - Should this person be copied on all emails sent by the HRPO office to the PI/POC? Study team members with edit rights will automatically receive all emails:

○ Yes ● **No**

5 * Does this study team member have a potential conflict of interest, financial or otherwise, related to this research?

○ Yes ● **No**

6 * Briefly describe experience conducting research and knowledge of the local study sites, culture, and society:
Dr. Krupnick has extensive research and clinical experience with this drug.

Add a Team Member

1 * Select Team Member:
Christopher Thomas

2 Research Role:
Research Team Member

3 * Edit Rights - Should this person be allowed full edit rights to the submission, including: editing the online forms and the ability to execute activities? Note - a person with edit rights will automatically be added to the CC list and will receive all emails regarding this protocol, even if the answer to #4 below is No.

○ Yes ● **No**

4 * CC on Email Correspondence - Should this person be copied on all emails sent by the HRPO office to the PI/POC? Study team members with edit rights will automatically receive all emails:

○ Yes ● **No**

5 * Does this study team member have a potential conflict of interest, financial or otherwise, related to this research?

○ Yes ● **No**

6 * Briefly describe experience conducting research and knowledge of the local study sites, culture, and society:
research team member
