# Supplementary material for: Regadenoson for the treatment of COVID-19: A five case clinical series and mouse studies
Source: PLoS One. 2023 Aug 11;18(8):e0288920. doi: 10.1371/journal.pone.0288920 (PMC10420352; doi:10.1371/journal.pone.0288920)
Supplement: Attachment — Submitted filename: PLOS Response to Reviewers Lau June 2023.docx [file pone.0288920.s007.docx]

June 2, 2023

Emily Chenette

Editor in Chief

PLOS ONE

Dear Dr. Chenette,

Although we were disappointed that our manuscript was not reviewed with greater alacrity, we do appreciate that you, in your capacity of Editor in Chief of PLOS ONE have taken it upon yourself to handle it, and that you and the reviewer found that previous concerns were addressed excepting one minor issue that has been rectified in this revision. Along with the revised manuscript we have also included some of our experimental protocols in protocols.io to ease the ability of others to reproduce our findings.

Although the significance of this study has waned due to the reduced incidence of Covid-19, the findings still have potential significance in providing improved understanding of the mechanism by which viruses elicit cytokine storm, and the potential targeting of the adenosine A2A receptor for the treatment of lung inflammation caused by viruses, other pathogens, transplantation, or trauma.

**Editor Q1**: Is the manuscript now acceptable for publication?

**Reviewer**: no response

**Author response**: Please see response to Comment 3.

**Editor Q2**: Is the manuscript technically sound, and do the data support the conclusions?

**Reviewer:** yes

**Author response**: We appreciate that the reviewer found that the experiments had been conducted rigorously.

**Editor Q3:** Has the statistical analysis been performed appropriately and rigorously?

**Reviewer:** I Don't Know.

**Author response**: We have elaborated on statistical methods used in the figure legends.

**Editor Q4**: Have the authors made all data underlying the findings in their manuscript fully available?

**Reviewer:** No

**Author response**: To comply with then PLOS ONE data policy that requires authors to make all data underlying the findings described in their manuscript fully available, we have now added supplemental data tables including all of the data used for the figures.

**Editor Q5**: Is the manuscript presented in an intelligible fashion and written in standard English?

**Reviewer:** yes

**Reviewer Q6:** All my comments have been addressed but regarding my question and your answer "The two-way ANOVA under figure 2 is not mentioned under methods. A two-way ANOVA analysis is not appropriate since it does not handle the repeated measures over time. Please double check which analysis that was performed or do a correct one. We can clearly see that there will be no effect on blood pressure but still the method for the statistical comparison should be correct.

**Author response**: Thank you very much. Our statistician has reviewed and changed to repeated measures applied to changes from baseline. We have updated the manuscript appropriately with statistical explanations.

Sincerely,

Christine L. Lau, M.D., MBA

Robert W. Buxton Professor

Chair, Department of Surgery

University of Maryland School of Medicine
